# The Combination of Antibiotic and Non-Antibiotic Compounds Improves Antibiotic Efficacy against Multidrug-Resistant Bacteria

**DOI:** 10.3390/ijms242015493

**Published:** 2023-10-23

**Authors:** Gang Xiao, Jiyun Li, Zhiliang Sun

**Affiliations:** College of Veterinary Medicine, Hunan Agricultural University, Changsha 410128, China; xiaogang2020@stu.hunau.edu.cn (G.X.); lijiyuny@foxmail.com (J.L.)

**Keywords:** multidrug-resistant bacteria, non-antibiotic compounds, antimicrobial mechanism, synergy

## Abstract

Bacterial antibiotic resistance, especially the emergence of multidrug-resistant (MDR) strains, urgently requires the development of effective treatment strategies. It is always of interest to delve into the mechanisms of resistance to current antibiotics and target them to promote the efficacy of existing antibiotics. In recent years, non-antibiotic compounds have played an important auxiliary role in improving the efficacy of antibiotics and promoting the treatment of drug-resistant bacteria. The combination of non-antibiotic compounds with antibiotics is considered a promising strategy against MDR bacteria. In this review, we first briefly summarize the main resistance mechanisms of current antibiotics. In addition, we propose several strategies to enhance antibiotic action based on resistance mechanisms. Then, the research progress of non-antibiotic compounds that can promote antibiotic-resistant bacteria through different mechanisms in recent years is also summarized. Finally, the development prospects and challenges of these non-antibiotic compounds in combination with antibiotics are discussed.

## 1. Introduction

With the unreasonable use of antibiotics, the problem of bacterial antibiotic resistance is becoming increasingly serious, which is a serious threat to human public health security. Antibiotic resistance is now the leading cause of death globally, with 1.27 million deaths having occurred directly from antibiotic-resistant infections and 4.95 million deaths occurring indirectly in 2019—far more than from other diseases such as acquired immune deficiency syndrome (AIDS) or malaria [1]. In addition, it is estimated that antibiotic-resistant bacteria will cause 10 million deaths per year and an economic loss of 100 trillion USD by 2050 [2]. Worryingly, new horizontal transmission-resistant genes and variants such as mobile colistin resistance (mcr-1) [3] are still being discovered, which further makes it difficult to treat and control gram-negative resistant bacteria. If left uncontrolled, the proliferation of AMR has the potential to render numerous bacterial pathogens significantly more lethal in the future than in their current state. This necessitates the exploration of novel alternative strategies to combat resistance.

Currently, strategies employed to combat bacterial antibiotic resistance include researching novel antimicrobial agents, the semisynthetic derivatization of existing antibiotics, screening for antibiotic alternatives, and prolonging the efficacy of existing antibiotics. Despite the recent FDA approval of new antibacterial drugs [4], developing novel antibiotics with unique targets, particularly against gram-negative bacteria, remains challenging. Moreover, the pace of new antibacterial drug development lags significantly behind that of resistant bacterial evolution [5]. Bacteria always develop resistance to any therapy introduced that relies solely on antibacterial mechanisms, and significant resistance can emerge in as short a period as a few months to a few years after the introduction of a new antibiotic into the clinic [6]. In spite of extensive efforts to identify alternatives to antibiotics, there are few viable options that can fully supplant antibiotics. In clinical practice, the use of antibiotics remains the primary choice for treating human and animal diseases. Therefore, considering the current difficulties and challenges, exploring novel approaches to prolong the efficacy of existing antibiotics could be a promising direction to pursue.

Combination therapies are considered a potentially promising strategy to combat antibiotic-resistant bacteria [7,8]. The additional stress of the combination may be more effective than either alone, which is a logic that supports the practice of combination therapy as a therapeutic strategy against MDR infections [7,8]. These combinations include antibiotic–antibiotic combinations, non-antibiotic–non-antibiotic combinations, and antibiotic–non-antibiotic combinations. Combinations of antibiotics and antibiotics have been used clinically and proven to be effective, such as the combination of trimethoprim and sulfamethoxazole [9], which was approved for use many years ago. However, the drawback of this combination lies in its potential to augment exposure to unnecessary antibiotics during usage, thereby amplifying bacterial resistance [10]. Non-antibiotic–non-antibiotic combination is an unexplored area. In addition, the therapeutic efficacy of the two non-antibiotic compounds in vivo, especially in the presence of complex body fluids, remains an unknown concern [10]. Therefore, antibiotic and non-antibiotic compound combinations are considered to be the most promising strategy [8]. The triumph of amoxicillin/clavulanate potassium serves as a testament to the feasibility of such a combination [11]. This non-antibiotic preparation showed very weak or even no antibacterial effect when used alone but could significantly enhance the activity of antibiotics when used in combination with antibiotics. This approach holds great promise as it has the potential to curtail antibiotic usage, mitigate resistance development, and, thus, prolong the efficacy of antibiotics.

In this review, we initially discussed the various mechanisms of bacterial drug resistance. Subsequently, our focus is on describing the situation and mechanism behind non-antibiotic compounds to enhance antibiotic activity, including plant-derived active ingredients, antimicrobial peptides (AMPs), metabolites, and phages. Finally, we analyze the feasibility and challenges associated with these combinations while also providing practical recommendations.

## 2. Mechanisms of Antibiotic Resistance in Bacteria

**Figure 1:**
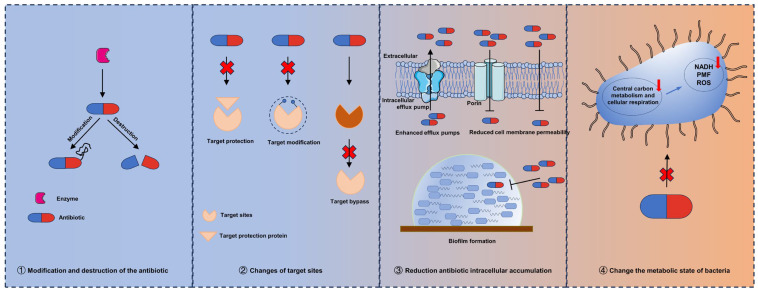
Overview of the molecular mechanisms of antibiotic resistance.

Studies have shown that bacterial resistance to antibiotics is actually the result of bacterial evolution [12,13]. Resistance genes developed by bacteria to antibiotics were present before the advent of antibiotics [14,15]. Currently, bacterial resistance to antibiotics can be chromosome-mediated or extrachromosomal mobile element-mediated. Antibiotic resistance can be achieved through a variety of mechanisms, including through the modification and destruction of the antibiotic, changes in target sites, a reduction in antibiotic intracellular accumulation, and through changing the metabolic state of bacteria (Figure 1). Understanding the mechanism of bacterial resistance is the premise and key to finding new ways to inhibit or reverse bacterial resistance.

### 2.1. Modification and Destruction of the Antibiotic

The production of specific enzymes by bacteria to alter and destroy antibiotics [16] is the main way for bacteria to cope with antibiotics, making them unable to work properly, and this is also one of the important mechanisms leading to antibiotic resistance. To date, a wide variety of resistance enzymes can degrade or modify different classes of antibiotics, including β-lactams, carbapenems, aminoglycosides, fluoroquinolones, tetracyclines, and macrolides.

β-lactamase is an important drug-resistance enzyme commonly produced in gram-negative bacteria. It is composed of serine-β-lactamase and metal-β-lactamase. β-lactamases inactivate β-lactam antibiotics by disrupting the β-lactam ring and changing the conformation of the antibiotic [17]. Extended-spectrum beta-lactamases (ESBLs) are one of the most relevant β-lactamases at present; they can hydrolyze a variety of antibiotics such as cefotaxime, ceftazidime, and aztreonam, as well as endow bacteria with resistance to a variety of β-lactam antibiotics. Metal-β-lactamase has a wide substrate spectrum and widely hydrolyzes all β-lactam antibiotics except monocyclic β-lactam antibiotics, and its activity requires metal ions (Zn) to be mediated, which is not inhibited by existing lactase inhibitors [18,19,20]. Moreover, it has a diverse structure and mechanism of action, making it difficult to overcome its mediated antibiotic resistance. New Delhi metalloproteinase-1 (NDM-1) is a newly discovered metalloenzyme that can render carbapenems and other β-lactam antibiotics such as penicillin ineffective, making bacteria extensively resistant to most antibiotics, including β-lactams, carbapenems, aminoglycosides, macrolides, and quinolones, and is sensitive only to polymyxin and tigecycline [21,22]. Therefore, NDM-1-producing bacteria are also called super bacteria. In addition, the NDM-1 gene is located on bacterial plasmids and is able to transmit horizontally among microorganisms, making NDM-1 widespread and capable of increasing the difficulty of prevention and control [23]. Of concern, when bacteria co-carry various extended-spectrum β-lactamases and carbapenemases [24,25], it can lead to resistance to almost all β-lactam antibiotics.

In addition to the direct destruction of antibiotics, the modification of antibiotics is also an important way for bacteria to develop drug resistance [26]. One typical example is aminoglycoside antibiotics, which contain numerous exposed hydroxyl groups and amino groups that are easy to modify [27,28]. The weak binding of modified aminoglycoside antibiotics to ribosomes weakens the antibacterial effect of antibiotics and promotes bacterial resistance. Common aminoglycoside-modifying enzymes include acetyltransferases, phosphorylatases, adenylyases, and nucleosidases [28,29]. The genes responsible for encoding aminoglycoside-modifying enzymes are usually found in plasmids and transposons, although they can also be found on chromosomes [27]. Recently, Bordeleau et al. [30] discovered a new aminoglycoside-modifying enzyme, APMA, which is an acetyltransferase capable of inactivating ampramycin. It is worrisome that aminoglycoside-modifying enzymes are often associated with ESBLs, leading to multidrug resistance. In addition, antibiotic-modifying enzymes have been identified for several antibiotics, including aminoglycosides, macrolides, rifamycins, streptogramins, lincosamides, and phenicols [31]. Nucleotidyltransferase encoded by the *lnu* gene modifies lincomycin by adding phospho-containing groups to the antibiotic [32]. Erythromycin esterase and macrolide 2′-phosphotransferase, produced by Enterobacteriaceae, prevent macrolide antibiotics from binding to the 50s ribosome by destroying the lipophilic ring of the tetracyclic macrolide, leading to bacterial resistance [33].

### 2.2. Changes in Target Sites

Another way in which bacteria evolve to resist antibiotics is by changing the target site to develop resistance [34,35]. There are several ways for bacteria to make an antibiotic unable to bind to the target site so that the antibiotic cannot work on the bacteria and, therefore, drug resistance, including the mutation of the gene encoding the target, the change of the target by the enzyme, and the target’s bypass [31,36]. A single point mutation in the *ropB* gene in *Escherichia coli (E. coli)*, which encodes the RNA polymerase, can result in high rifampicin resistance [37,38]. Resistance to fluoroquinolones is caused by mutations in the genes encoding DNA gyrase and topoisomerase IV, which are essential for DNA replication. In general, the coexistence of several mutations is more likely to cause a high degree of drug resistance. For example, four mutations in penicillin-binding protein PBP5 are often found in *Enterococcus faecium* with high drug resistance, but the mutation of any one site alone cannot cause a high degree of drug resistance: only the simultaneous occurrence of four sites can lead to a high degree of drug resistance.

In addition, the modification of the target of antibiotic action reduces antibiotic binding. Macrolide antibiotics prevent bacterial protein synthesis by reversibly binding the peptidyl-tRNA binding site of the 50s ribosomal subunit, preventing the translocation of newly synthesized peptidyl-tRNA molecules from the acceptor site to the donor site. Ribosomal methylation modification mediated by *erm* genes is the main way for bacteria to block the action of macrolides, and several common ERMs include *erm(A)* and *erm(C)* in *Staphylococcus* and *erm(B)* in *Pneumococcus* and *Enterococcus* [39,40]. At present, the most widely studied colistin resistance gene, *mcr*, encodes phosphoethanolamine transferase, which adds phosphoethanolamine to lipid A, reduces the negative charge of lipopolysaccharide to reduce the binding of colistin, and mediates bacterial resistance to colistin [3]. In addition, chloramphenicol–florfenicol resistance *(CFR)* to methyltransferase, which is encoded by the *CFR* gene carried by the plasmid, can specifically methylate A2503 in 23S rRNA, thus giving bacteria resistance to linezolid [41,42].

In addition, target bypass is a strategy employed to make the original target redundant by generating alternative pathways to bypass antibiotics. The binding of methicillin to the target PBPS prevents the synthesis of the cell wall to kill bacteria. However, the replacement protein PBP2a, which is encoded by the *mecA* gene of *Staphylococcus aureus (S. aureus)*, does not inhibit the cell wall when methicillin binds to this replacement target, ultimately leading to the formation of methicillin-resistant *S. aureus* (MRSA) [43,44]. In *E. coli*, the peptidoglycan crosslinking reaction that occurs to form the cell wall is primarily carried out by penicillin-binding proteins (PBPs), the target of β-lactam antibiotics, which catalyze D, D-transpeptidase activity. Recently, Caveney et al. [45] found that an alternative cross-linking mechanism mediated by L, D-transpeptidase YcbB can lead to a bypass of the PBP-mediated D, D-transpeptidase action, resulting in bacterial resistance to β-lactam antibiotics.

### 2.3. Reduction in Antibiotic Intracellular Accumulation

Some antibiotics have their targets inside the cell or inside the cell membrane, which they must cross before they can work. In view of this, clever bacteria find ways to reduce antibiotic target contact, such as biofilm formation, reduced cell membrane permeability, and enhanced efflux pumps. Due to differences in the composition of the outer membrane, gram-negative bacteria form a natural permeability barrier; therefore, the permeability of antibiotics in gram-negative bacteria is lower than that in gram-positive bacteria, especially for lipophilic antibiotics. However, there are special proteins on the outer membrane of gram-negative bacteria, such as porins [46], which can allow the passage of some hydrophilic substances or nutrients, such as OmpF and OmpC in *E. coli*, ompD in *salmonella*, OmpK35 and OmpK36 in *Klebsiella pneumoniae* (*K. pneumoniae*) [47,48], and OprD in *Pseudomonas aeruginosa* (*P. aeruginosa*). Bacteria are made resistant to these antibiotics by down-regulating the expression of these proteins to reduce bacterial permeability. In addition, the relationship between the loss or down-regulation of porin expression and the development of drug resistance is complex, and it is often accompanied by the expression of efflux pumps.

In addition to preventing antibiotics from entering the cell, the efflux of intracellular antibiotics is also an important way to cause bacterial resistance. The efflux pumps on the surface of the bacterial cell membrane, which actively expel antibiotics from the cell, play an essential role in bacterial drug resistance [46,49]. The antimicrobial efflux pumps in bacteria can be divided into five main superfamilies [50,51]: ① ATP-binding cassette transporters (ABC family); ② the major facilitator superfamily (MFS family); ③ resistant-nodulation-division families (RND family); ④ small multidrug resistance families (SMR family); ⑤ multidrug and toxic compound extrusion (MATE) families. Among them, the RND family is one of the most extensively studied efflux pumps due to the wide range of substrates found in almost all gram-negative strains [52]. The active efflux pump in gram-negative bacteria consists of three components: an outer membrane protein, a membrane fusion protein, and an efflux protein or transporter on the inner membrane. The RND efflux pump AcrAB-TolC, which is composed of a periplasmic fusion protein AcrA, a plasma membrane transporter AcrB, and an outer membrane channel protein TolC, plays an important role in the process of multidrug resistance in *E. coli* [53]. Before the drug acts on the intracellular target site, it binds to the plasma membrane transporter AcrB and is expelled from the cell through AcrA and the outer membrane channel TolC. AcrB has very low substrate specificity; therefore, many structurally diverse compounds can act as substrates, which is the reason for its multidrug resistance. More importantly, AcrAB-TolC plays a central role in the acquisition of acquired drug resistance conferred by the resistant plasmid, which facilitates and conditions the spread of genes that encode multidrug-resistant efflux pumps [53].

The overexpression of a drug efflux pump is an important mechanism of multidrug resistance in *Acinetobacter baumannii* (*A. baumannii*). AdeABC is the first reported and most studied RND efflux pump in *A. baumannii*. AdeABC is associated with aminoglycoside resistance and has a certain efflux effect on quinolones and tetracyclines. In addition, AdeABC can work with carbapenemases or outer membrane proteins to mediate carbapenem resistance. In addition, efflux pumps also play an important role in the resistance of *P. aeruginosa* to antibiotics. For example, MexE-MexF-OprN is associated with the efflux of carbapenems, fluoroquinolones, and chloramphenicol antibiotics [54,55].

In addition, bacterial biofilm is also a natural barrier that restricts the entry of antibiotics into bacteria. The formation of bacterial biofilm not only acts as a barrier but also assists some enzymes, such as β-lactamase, in destroying antibiotics, thereby increasing bacterial resistance. In addition, because some of the substances that form biofilms are positively charged, it can add a charge barrier to some positively charged antibiotics, such as aminoglycosides.

### 2.4. Change the Metabolic State of Bacteria

A large body of evidence suggests that bacterial metabolism is closely related to antibiotic potency [56]. Bactericidal antibiotic treatment disrupts cell homeostasis and results in increased ATP demand, increased metabolic burden, and then, gradually, increased toxic metabolic by-products, thus inducing cellular death [57,58]. Bacteria with reduced metabolism are resistant or tolerant to many classes of antibiotics, and increased drug sensitivity is associated with enhanced metabolism [58,59]. Lopatkin et al. found that genes associated with central carbon and energy metabolism are associated with antibiotic resistance. These metabolic alterations result in lower basal respiration and thus prevent the induction of antibiotic-mediated tricarboxylic acid cycle (TCA cycle) activity, thereby evading metabolic toxicity and minimizing drug lethality [60]. Genetically increasing the basal respiration rate of *E. coli* increases the efficiency of bactericidal antibiotics against wild-type cells [61]. In addition, quiescent or auxotrophic bacteria can exhibit resistance to a variety of antibiotics. Studies have shown that *E. coli* under starvation conditions will lead to an excessive production of guanosine tetraphosphate ppGpp, which hinders the synthesis of peptidoglycans and phospholipids and leads to bacterial resistance to penicillin antibiotics [62]. The development of metabolomics has provided a useful technique for studying the metabolic state of drug-resistant bacteria. Through metabolomics studies on MDR bacteria, it was found that changes in the glucose metabolism and amino acid metabolism of bacteria can disturb the central metabolic pathway TCA cycle and affect the electron transfer in the respiratory chain, which can affect the sensitivity of bacteria to antibiotics and cause bacterial tolerance or drug resistance [63,64]. Peng et al. [65] compared the metabolomics of kanamycin-resistant *Edwardes fluminata* and sensitive *E. dwardes* and found that the MDR bacteria had defects in the central metabolic pathways, especially in glucose metabolism and amino acid metabolism, which was the same as the previous findings in resistant *Stenotrophomonas maltophilia* [66] and *P. aeruginosa* [67]. Furthermore, the exogenous addition of glucose and alanine, in combination with antibiotics, could restore the kanamycin sensitivity of the resistant bacteria. This emphasizes that the metabolic status of bacteria is closely related to its resistance to antibiotics. In summary, the physiological metabolism of bacteria can affect their sensitivity to antibiotics, but the physiological metabolic process of bacteria is extremely complex and affected by many factors. Therefore, it is necessary to further study the mechanism of the relationship between bacterial physiological metabolism and antibiotic sensitivity.

## 3. Strategies to Enhance the Action of Antibiotics against Resistant Bacteria

**Figure 2:**
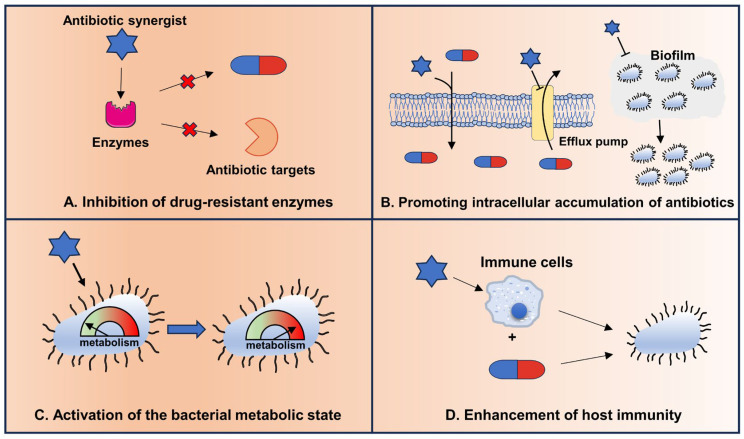
Strategies to enhance the efficacy of antibiotics against resistant bacteria.

Therefore, we propose that it is theoretically feasible to reverse the antibiotic resistance of bacteria by inhibiting the activity of drug-resistant enzymes, increasing the intracellular accumulation of antibiotics, activating the metabolic state, and enhancing host immunity (Figure 2).

### 3.1. Inhibition of Drug-Resistant Enzymes

Bacteria produce specific enzymes to alter and destroy antibiotics or change the target of antibiotics, which is one of the important mechanisms leading to antibiotic resistance. Therefore, the antibiotic sensitivity of resistant bacteria will be restored by inhibiting the action of these enzymes. At present, the most widely studied and only clinically available ones are β-lactamase inhibitors. A well-known example is amoxicillin clavulanate potassium, the first FDA-approved combination of an antibiotic and a non-antibiotic substance for clinical use. The irreversible inactivation of serine β-lactamase by clavulanic acid has greatly enhanced the action of β-lactam antibiotics [68,69], making them the drug of choice for the treatment of most bacterial diseases. With the successful marketing of combination drugs such as clavulanate potassium and amoxicillin, more and more β-lactamase inhibitors have been discovered or approved for application, including diazabicyclooctanes (DABCOs) [70], boronic acid vaborbactam [71], and ETX2514 [72]. Nonetheless, the fact that no metallo-β-lactamase-antibiotic combinations have been approved for clinical use is concerning, given the wide variety of β-lactamases and, in particular, the prevalence of pathogens with metallo-β-lactamases with extremely high and rapid incidence. To combat these, there is an urgent need to explore novel inhibitors of metallo-β-lactamase. Fortunately, a fungal natural product, aspergilomycin A, was screened and found to be an NDM-1 inhibitor and showed significant effects in vitro and in vivo when combined with meropenem [73]. Subsequently, numerous metallo-β-lactamase inhibitors were discovered, including isoliquiritin [74], sulfamoyl heteroarylcarboxylic acids [75], fisetin [76], and ANT2681 [77].

In addition, some enzymes inactivate antibiotics by modifying them. Thus, the inhibition or inactivation of these modifying enzymes is important. Zhu et al. [78] used the method of UPLC-QTOF MS to determine that pyrimidinyl indole derivatives have inhibitory effects on both aminoglycoside phosphotransferase and aminoglycoside acetyltransferase, which are effective inhibitors of aminoglycoside resistance enzymes. Boehr et al. [79] found that the cationic peptide-bovine antimicrobial peptide indolicidin and its synthetic analogues have inhibitory effects on both aminoglycoside phosphotransferase and aminoglycoside acetyltransferase, which are effective broad-spectrum inhibitors of aminoglycoside-resistant enzymes.

### 3.2. Increased Intracellular Accumulation of Antibiotics

For some intracellular-acting antibiotics, a certain dose of intracellular concentration is required to exert an effect; therefore, it is important to enhance the accumulation of antibiotics in bacterial cells. Especially for gram-negative bacteria, the complex outer membrane structure and various mechanisms that reduce the accumulation of intracellular antibiotics seriously hinder the passage of some antibiotics and reduce their potency. Therefore, promoting intracellular antibiotic concentrations is important to increase antibiotic potency. The intracellular accumulation of antibiotics can be improved through several pathways, including reducing drug efflux, improving cell membrane permeability, and reducing biofilm formation.

As previously described, the RND efflux pump is an MDR efflux pump that effluxes a variety of antibiotics, causing multidrug resistance in *Enterobacteriaceae* and *P. aeruginosa*. Recently, Ple et al. [80] identified a series of pyridine–piperazine-based RND efflux pump inhibitors that sensitize *E. coli* to antibiotics by binding to unique locations on the transmembrane domain of the AcrB transporter, thereby inhibiting antibiotic efflux. Given that proton motive force (PMF) powers the efflux pump [81], the inhibition of PMF in bacteria to block the energy source of the efflux pump can also be achieved to inhibit the efflux pump, thereby enhancing the effect of antibiotic action. Cationic AMPs have the potential to disperse transmembrane PMF. C12(ω7)K-β12 [82] is a small cationic lipopeptide that can enhance the activity of tetracycline and erythromycin against *E. coli* by removing the proton driving force required for the active efflux of bacteria through transient membrane depolarization. In addition, some substances can compete with antibiotics to bind to the active site of the bacterial efflux pump, resulting in the retention of the antibiotic in the cell, which, in turn, increases the antibiotic effect. Phenylalanine-arginine beta-naphthylamide (PAβN), as a competitive inhibitor, prevents the efflux of the bacterial efflux pump by binding to the substrate-binding pocket of the efflux pump to block the efflux of antibiotics, resulting in the potentiation of their activity [83,84,85,86]. Alternatively, due to the close position of the binding site, efflux pump inhibitor binding may also create a steric hindrance that impairs antibiotic binding at its affinity site. Currently, PAβN has been studied in a variety of gram-negative bacteria, including *E. coli*, *K. pneumoniae*, *A. baumannii*, *S. enterica*, and *P. aeruginosa*, and it has been shown to potentiate the activity of different antibiotics by acting on the various efflux pumps [84,87,88].

For gram-negative bacteria, the presence of a complex structure of the outer membrane causes the bacteria to become intrinsically resistant to many antibiotics. More seriously, high levels of multidrug resistance, formed by the dual holding effect of the diminished permeability of the outer membrane and efflux of drugs from the efflux pump, are even more damaging. Therefore, increasing the permeability of the outer membrane helps to improve the sensitivity of gram-negative bacteria to various antibiotics. Divalent cations cross-link LPS molecules by forming ionic bridges with the negatively charged phosphate groups of lipid A and are indispensable for the integrity of the outer membrane of gram-negative bacteria [89], suggesting that the disruption of these structures to increase outer membrane permeability contributes to the susceptibility of gram-negative bacteria to several antibiotics. Various molecules can disrupt the physical structure of the outer membrane of gram-negative bacteria by removing or competing with divalent ions, thereby breaking the cross-linking structure between the divalent cation and the LPS molecule. Such molecules include charge-containing small-molecular-weight drugs [90], cationic AMPs [91], chelating agents, and cationic polymers [92]. Stokes et al. [90] screened 1440 approved drugs and found that pentamidine could increase the susceptibility of *E. coli* and *A. baumannii* to novobiocin and rifampicin by increasing the permeability of the outer membrane through the disrupted cationic bridges of the maintaining LPS molecules. As for AMPs, the prime example is colistin [93], which binds to LPS much more strongly than divalent magnesium or calcium ions; therefore, it competitively displaces these divalent ions, weakening and releasing the LPS molecules to form permeable pores in the outer membrane, which not only fights the bacteria but also works synergistically with other antibiotics, such as carbapenems. In addition, a cation-blocking β-peptide (PAS8-b-PDM12) reported by Si et al. [94] was found to reverse the resistance of carbapenem-resistant, gram-negative bacteria to a variety of antibiotics through two different mechanisms of action: destroying the integrity of the bacterial outer membrane and dissipating the transmembrane electrochemical potential to disable the efflux pump system.

In addition, biofilms with bacterial-secreted polysaccharides and proteins, which provide a physical barrier, an altered chemical microenvironment, and a dormant metabolic state for bacteria, greatly increase the odds of antibiotic resistance. Thus, the inhibition or disruption of biofilm formation contributes to the action of antibiotics. Several of the non-antibiotic anti-biofilm compounds identified are mainly derived from natural products, synthetic compounds, chelating agents, metabolites, and AMPs [95,96,97]. Quorum sensing (QS) is a process whereby the expression of certain genes in bacteria is regulated by signaling molecules associated with the density of the population; moreover, it plays a crucial role in regulating the formation of the bacterial biofilm [98]. In addition, c-di-GMP has also been proven to be closely associated with the formation of the gram-negative bacterial biofilm. It has been shown that high levels of c-di-GMP promote biofilm formation by promoting polysaccharide biosynthesis, while low levels of c-di-GMP cause biofilm dispersion by enhancing flagellar formation and bacterial dispersion [99]. Thus, QS modulation and c-di-GMP modification became targets for anti-biofilm formation. Chen et al. [100] identified a quorum sensing inhibitor (QSI), norharmane, that strongly inhibits the biofilm formation of MDR *P. aeruginosa*. Moreover, norharmane is synergistic with polymyxin B. Norharmane improves the activity of polymyxin B against MDR *P. aeruginosa* in vitro and in vivo. Recently, it has been shown that ebselen inhibits *ESBL-E. coli* resistance to β-lactam antibiotics by inhibiting the diguanylate cyclase DgcM and modulating c-di-GMP levels [101]. In conclusion, these examples demonstrate that the increased intracellular accumulation of antibiotics enhances the effect of antibiotics against MDR bacteria. Certainly, it would be better to explore substances that simultaneously inhibit the efflux pump, increase permeability, and inhibit biofilm formation than those that have only a single effect. For example, baicalein [102] can bind to phospholipids on the cytoplasmic membrane and lipopolysaccharide on the outer membrane of gram-negative bacteria to cause membrane rupture and enhance bacterial permeability. It can also inhibit the activity of the multidrug efflux pump and the formation of biofilm, thereby enhancing the efficacy of doxycycline.

### 3.3. Activation of the Bacterial Metabolic State

The metabolic state of bacteria can affect the efficacy of antibiotics, suggesting that metabolic regulation can improve the efficacy of antibiotics [59]. The resistant bacteria with weak metabolisms can reverse their sensitivity to antibiotics by using exogenous substances to improve their metabolism. The activation of bacterial metabolism can improve the sensitivity of drug-resistant bacteria to antibiotics by promoting the intracellular accumulation of antibiotics. For example, Peng et al. [65] showed that exogenous alanine and glucose restored the sensitivity of drug-resistant bacteria to kanamycin by promoting the uptake of antibiotics by bacteria by promoting the TCA cycle and increasing the production of Nicotinamide adenine dinucleotide (NADH) and PMF. In addition, Allison et al. [103] found that adding different metabolites (glucose, mannitol, fructose, and pyruvate) to the medium enhanced the effect of gentamicin on *S. aureus* and *E. coli*. Further studies have shown that these four carbon sources stimulate the production of PMF in the respiratory chain through catabolism to produce NADH, thus promoting the uptake of aminoglycoside antibiotics by cells and enhancing the killing effect of antibiotics [103], which further support the aforementioned point of view. Reactive oxygen species (ROS) production is a general mechanism by which bactericidal antibiotics kill bacteria [104,105,106,107,108]. In brief, the interaction of antibiotics with specific targets activates the TCA cycle and the electron transport chain, leading to the generation of free radicals that damage bacterial DNA, lipids, and proteins [109]. In view of this, increasing ROS production by regulating metabolic networks may enhance antibiotic activity [106]. Recently, Wu et al. [110] found that L-serine is an ROS enhancer that can produce synergistic killing effects against macrolide-resistant *Streptococcus suis* in vitro and in vivo when combined with macrolide antibiotics. Further studies found that L-serine inhibited the production of intracellular H_2_S, reduced the production of Fe-S clusters, and restored the intracellular Fenton reaction, which, eventually, caused an increase in the level of bacterial endogenous reactive oxygen species, leading to intracellular DNA damage and bacterial death. Bacterial respiration is a potential source of ROS, suggesting that the modulation of cellular respiration is also very important for increasing the potency of antibiotics. Previous studies have shown that the inhibition of cellular respiration can increase tolerance or resistance to antibiotics; meanwhile, increasing cellular respiration can restore sensitivity to antibiotics [111]. For example, Liu et al. found that cysteine [112] and thymine [113] enhanced the activity of antimicrobial antibiotics by up-regulating the bacterial TCA cycle, respiration, and oxidative damage. Su et al. revealed that exogenous glutamate reversed the phenotype of antibiotic resistance in bacteria by stimulating pyruvate cycle (*P* cycle) metabolic flux and enhancing energy production in resistant bacteria, emphasizing the role of cellular respiration and energy production in reversing bacterial antibiotic resistance [114]. Interestingly, the strategy of activating bacterial metabolism against drug-resistant bacteria is not only effective in vitro but also in vivo, suggesting that this strategy holds promise for clinical application. For example, glutamine was inhibited in multidrug-resistant uropathogenic *E. coli*. The exogenous addition of glutamine promotes the killing of multidrug-resistant uropathogenic *E. coli* through several antibiotics in vitro, and, in vivo, it not only inhibits biofilm formation in these bacteria but also increases the antibiotic inhibition of systemic infections caused by these bacteria in mice [115]. Together, these studies suggest that the activation of metabolism can enhance the killing effect of antibiotics against tolerant or resistant bacteria in different physiological states.

### 3.4. Enhancement of Host Immunity

The host’s innate immune system is an important defense line against bacterial infection, suggesting that the regulation of the host immune system may be an important way to enhance the effect of antibiotics on MDR bacterial infection. Although some substances have no direct antibacterial effect, they have been shown to have immunomodulatory effects, which can produce synergistic effects when combined with antibiotics to increase their potency. In a previous study, Chen et al. [116] used metabolomics to screen L-valine, a metabolic marker, in mice that survived infection with *K. pneumoniae*. Furthermore, L-valine was found to enhance the clearance of *K. pneumoniae* and *E. coli* by activating the p13k/akt1 pathway to increase macrophage phagocytosis and NO production. Jiang et al. [117] used metabolomics to screen maltose, a metabolic differential marker of zebrafish infected with levofloxacin-sensitive or levofloxacin-resistant *Vibrio alginolyticus* (*V. alginolyticus*). Further research showed that exogenous maltose enhanced the immune response of zebrafish to levofloxacin-resistant *V. alginolyticus* by increasing the production of host lysozyme, thereby improving the clearance of bacterial infection. Some AMPs affect the host immune system in various ways, including suppressing inflammation to prevent infection from triggering an excessive immune response leading to sepsis and inducing host cell-based antimicrobial activities such as increased phagocytosis. The (TPFI-2)-derived EDC34 peptide [118] has a strong immunomodulatory effect and can reduce the excessive inflammatory response caused by bacterial endotoxin. The peptide alone could not rescue the mice with sepsis caused by *P. aeruginosa*; however, when used in combination with ceftazidime, it could promote the formation of anaphylactotoxin C3a in the host, enhance the effect of antibiotics, and reduce the mortality of the model mice. In addition, some plant-derived active ingredients can also enhance the host’s immune system to promote the clearance of pathogen infections (Figure 2). For example, the synergistic antibacterial effect of the citrus flavonoid rutin and florfenicol on *Aeromonas hydrophila* (*A. hydrophila*) in vitro and in vivo [119]. The mechanism is that the rutin/florfenicol synergistic combination treatment of tilapia against *A. hydrophila* infection is achieved by improving blood cell count and anti-protease and lysozyme activities, as well as by reducing oxidative stress and pathological changes in tilapia to enhance host immunity. These examples suggest that, through the dual action of enhancing body immunity and antibiotic intervention, drug-resistant bacteria can be better cleared.

## 4. Combination Antibiotics and Non-Antibiotic Compounds

### 4.1. Combination Antibiotics and Plant-Derived Active Ingredients

**Figure 3:**
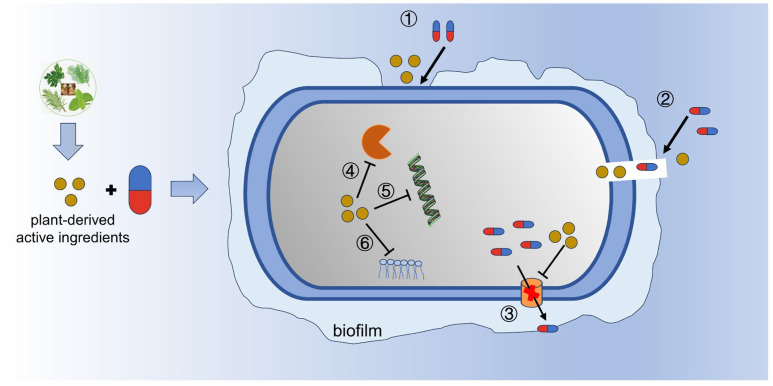
The synergistic action between plant-active ingredients and antibiotics: ① inhibit biofilm information; ② disrupt cell membrane; ③ inhibit efflux pumps; ④–⑥ inhibit the synthesis of DNA, proteins, and lipids.

Chinese herbs have a long history of being widely used in traditional medicine to treat infectious diseases. Active monomeric compounds from natural plants have the advantages of being less toxic, having fewer side effects, having more targets, and having less resistance. Its antibacterial effect is relatively weak compared with that of antibiotics. However, an increasing number of studies have shown that the active ingredients of natural plants can enhance the efficacy of antibiotics through a variety of different mechanisms, including the inhibition of biofilm formation, disruption of cell membranes, inhibition of the efflux pump, and inhibition of DNA, protein, and lipid synthesis (Figure 3). The pluripotency of phytochemicals can stimulate the antimicrobial activity of aminoglycosides, quinolones, macrolides, and tetracyclines [120]. Studies have shown that the combination of active compounds from natural plants and antibiotics often shows synergistic effects against MDR bacteria, including β-lactams, quinolones, aminoglycosides, tetracyclines, and glycopeptides [121]. These plant derivatives mainly include flavonoids, alkaloids, terpenoids, and phenols, as shown in Table 1 below. These plant active ingredients can interfere with membrane structure through the modification of bacterial cell membranes, which increases cell permeability and cellular leakage, resulting in a loss in ATP, the disruption of DNA, the inhibition of protein synthesis, DNA gyrase, QS, and biofilm formation [2,120,121]. Furthermore, Catteau et al. [122] found that ursolic acid/oleanolic acid extracted from the leaves of shea trees have a synergistic antibacterial effect when combined with ampicillin/oxacillin against MRSA by delocalizing PBP2 from the septal division site and interfering with peptidoglycan synthesis. Recently, Zhong et al. [123] screened three plant-derived flavonoids (catechol-type flavonoid-7,8-dihydroxyflavone, myricetin, and luteolin) and showed the synergistic effects they displayed when combined with colistin against MDR bacteria. The mechanism suggests that these flavonoids disrupt bacterial iron homeostasis by converting iron trivalent to ferrous forms and promoting colistin binding and membrane damage, which, in turn, promote the action of colistin against resistant bacteria. In addition, essential oils (EOs), a class of volatile small-molecule mixtures derived from plants, have been shown to possess potent antimicrobial activity. The composition of EOs is extremely complex, including terpene hydrocarbons, aromatic hydrocarbons, alcohols, aldehydes, ketones, ethers, esters, and phenols [124,125]. Due to the diverse and complex nature of their components, EOs can simultaneously exhibit activity against different bacterial targets. This multi-targeting property has a great advantage against multidrug-resistant bacteria compared to the single target of traditional antibiotics [126,127]. Based on this, plant EOs may have less potential for the development of microbial resistance [128,129]. In addition, in vitro and in vivo experiments have demonstrated synergistic effects between combinations of EOs and antibiotics [124,126,127]. The combination of EOs and antibiotics not only exerts multi-targeted antimicrobial activity but also effectively reduces or reverses microbial resistance, which may be an effective strategy to combat microbial resistance [124,130]. Studies have shown that the mechanisms of synergism between plant EOs and antibiotics mainly include increasing outer membrane permeability, inhibiting bacterial efflux pumps, and resisting group-sensing abilities. In addition, EOs have anti-inflammatory, antioxidant, and immunomodulatory effects, which suggests that EOs play an important role in promoting the treatment of multidrug-resistant bacterial infections [128]. These studies indicate that the combination of antibiotics with active compounds from natural plants provides a promising approach to combating drug-resistant bacteria.

### 4.2. Combination Antibiotics and Metabolites

**Figure 4:**
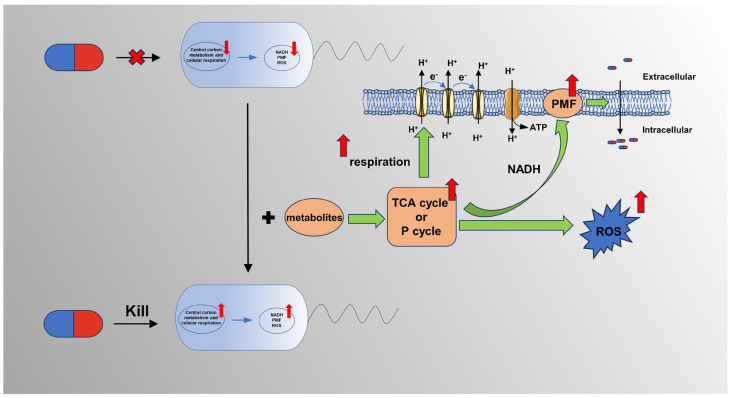
The synergistic action between bacterial metabolites and antibiotics: the combination of metabolites and antibiotics enhances the efficacy of antibiotics because exogenous metabolites stimulate the bacterial TCA cycle and *P* cycle, resulting in increased bacterial cell respiration, PMF, and ROS.

It is a new idea to repair metabolic deficiency through an exogenous increase in metabolites, but its combination with antibiotics can indeed increase the sensitivity of drug-resistant bacteria to antibiotics and prolong the life span of antibiotics. Numerous studies [169] have confirmed that the exogenous addition of metabolites such as those in the TCA cycle, amino acids, and nucleotides can increase the sensitivity of drug-resistant bacteria to antibiotics through different mechanisms (Figure 4). Additional examples are summarized in Table S1.

The TCA cycle is the central link of cellular energy metabolism, which is the hub of carbohydrate, fat, and amino acid metabolism. Numerous studies have shown that promoting the TCA cycle can change the metabolic state of bacteria, thereby improving the efficiency of antibiotics. Peng et al. found significant metabolic differences between kanamycin-resistant and -susceptible *Edwardella tard* (*E. tard*), with glucose and alanine abundances being suppressed in resistant strains. The combination of exogenous alanine or glucose with kanamycin restores susceptibility to kanamycin in MDR *E. tarda*. Further mechanistic studies showed that exogenous glucose or alanine promoted the TCA cycle by activating substrates, which, in turn, increased the production of NADH and PMF and then stimulated the uptake of antibiotics [65]. A similar mechanism was found in later studies, wherein exogenous low-abundance TCA metabolites were able to restore tobramycin sensitivity in *P. aeruginosa* [170]. In a later study, Su et al. further revealed that glutamate, another suppressed metabolite, could enhance the efficacy of aminoglycoside antibiotics, but the mechanism was not the same, indicating that exogenous glutamate could provide energy for bacterial respiration by regulating the flux of the *P* cycle, thereby reversing the sensitivity of *E. tarda* and *E. coli* to kanamycin [114].

In addition, nucleotide metabolism is an important metabolic process in organisms and has a variety of biological functions, including energy storage and metabolic and physiological regulation. Antibiotics are often able to destroy the nucleotide pool and affect nucleic acid metabolic pathways during the process of killing bacteria. Numerous studies have shown that nucleotides combined with antibiotics can enhance the sensitivity of bacteria to antibiotics [171]. Yang et al. [57] designed a “white box” biochemical screening, network modeling, and machine learning approach to screen different metabolites for the action of bactericidal antibiotics in *E. coli*, and the results showed that purine biosynthesis was involved in the lethal action of antibiotics. Further studies have shown that antibiotic-induced adenine limitation increases ATP demand, which improves central carbon metabolic activity and oxygen consumption, thereby enhancing the killing activity of antibiotics [172]. In addition, studies have shown that thymidine can enhance the killing effect of antibiotics on a variety of gram-negative bacteria by up-regulating bacterial metabolism, including increasing the TCA cycle and respiration, thereby promoting the production of ATP and ROS [113].

In addition, some evidence suggests that the combination of amino acids with antibiotics can enhance the activity of antibiotics by increasing PMF, up-regulating the P cycle, stimulating bacterial respiration, producing ROS, or stimulating the host immune response. For example, exogenous L-lysine can enhance PMF and stimulate the uptake of aminoglycosides by promoting the transmembrane proton gradient, which increases the sensitivity of gram-negative bacteria *A. baumannii*, *E. coli,* and *K. pneumoniae*, as well as gram-positive bacteria *Mycobacterium smeggy* to aminoglycosides [173]. L-leucine increases the sensitivity of drug-resistant *Salmonella* to salafloxacin by stimulating central carbon metabolism and increasing the levels of intracellular reactive oxygen species [174].

Interestingly, in vivo experiments [175,176,177] have shown that exogenous metabolites can regulate various physiological processes, including the immune response, antioxidant capacity, and inflammatory response, indicating that host metabolites may also play an important role in improving the efficacy of antibiotics [58,178]. For example, these different mechanisms indicate that adding exogenous metabolites to restore metabolic defects provides an attractive method to treat drug-resistant pathogens in combination with otherwise ineffective antibiotics [179]. Taken together, these studies suggest that the strategy of reprogramming metabolic pathways in resistant bacteria using metabolite molecules opens a promising avenue for extending the lifespan of antibiotics as well as for the development of novel antimicrobial therapies.

### 4.3. Combination Antibiotics and AMPs

**Figure 5:**
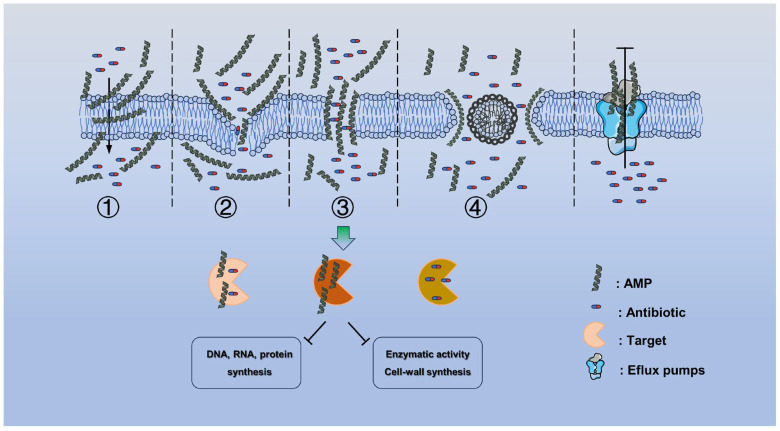
The underlying synergy mechanisms between AMPs and antibiotics: AMPs change membrane permeability through four membrane destruction mechanisms, including ① aggregate model; ② toroidal pore model; ③ barrel-stave model; ④ carpet model, which induces antibiotics to penetrate into cells, allowing them to reach and interact with targets in bacterial cells. In addition, AMPs block bacterial efflux pumps and increase intracellular antibiotic concentrations, thereby significantly improving the efficacy of conventional antibiotics.

AMPs are a class of short, cationic, and amphiphilic peptides that can be isolated from a variety of organisms and obtained using chemical synthesis. They exhibit broad-spectrum antimicrobial activity and immunomodulatory activity [180,181,182]. The main mechanism of action of antibacterial peptides is the rapid destruction of bacterial cell membrane structure. Several commonly accepted hypothetical models have been proposed to explain how AMPs damage cell membranes, including the carpet model, bucket plate model, annular hole model, and aggregation model (Figure 5 (①–④)) [183,184]. Both antibiotics and AMPs have antibacterial activity. However, antibiotics work primarily by interacting with specific molecular targets (e.g., cell wall synthesis, cell membrane, protein synthesis, and nucleic acid transcription and replication), which is different from the mode of action of AMPs. Given that two sufficiently different selection pressures are likely to be more effective than either alone and that antibiotics and antibacterials have the same purpose, it is possible that they work together to enhance each other’s effectiveness [183,184]. A growing number of AMPs were found to have synergistic effects with multiple antibiotics against MDR bacteria in vitro and in vivo (Table S2). The synergy mechanisms of AMPs and antibiotics (Figure 5): (1) Improve the uptake of antibiotics. When used in combination with antibiotics, AMPs can increase membrane permeability, enabling antibiotics that were blocked outside the cell to enter the cell and bind to the target [185]. In addition, AMPs can also increase antibiotics’ efficacy by blocking the efflux pump and reducing the pumping out of intracellular antibiotics. (2). Promote the binding of AMPs to the cell membrane; the increase in cell membrane permeability caused by AMP alone was quite slow, but the process of membrane disruption was quite rapid in the presence of certain antibiotics (e.g., gentamicin) [186]. These antibiotics enhance AMP selectivity by participating in the processes of lipid aggregation and membrane perturbation, thereby accelerating AMP integration into the lipid bilayer and/or its aggregation [186,187]. (3). Disturb bacterial metabolism. Some AMPs and antibiotics can affect bacterial metabolism by inhibiting protein synthesis, inhibiting nucleic acid (DNA and/or RNA) synthesis, and inhibiting enzyme activity, subsequently promoting their synergistic activity [184].

Therefore, combinations of antibiotics and AMPs are also potential therapeutic strategies for overcoming antibiotic resistance, improving bacteria-killing effects, and reducing toxicity and side effects [185]. This strategy can help reduce side effects and increase the selectivity of compounds while enhancing the permeability of bacterial membranes and reducing the efflux of antibiotic drugs, thereby inhibiting bacterial survival [188].

### 4.4. Combination Antibiotics and Metal-Based NPs

**Figure 6:**
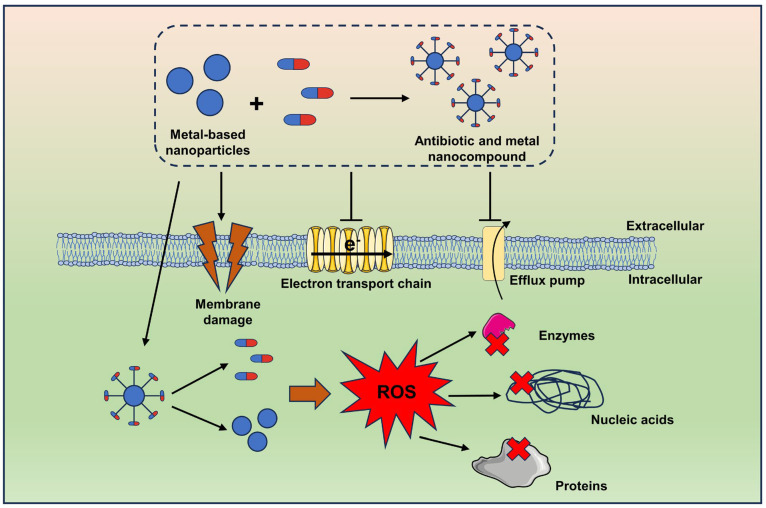
The synergistic action between metal-based NPs and antibiotics. This potential mechanism includes the disruption of the membrane structure, disruption of the electron transport chain, generation of ROS, and disruption of intracellular structures (nucleic acid, proteins, and enzymes).

Metals and metal oxides are known to possess antimicrobial activity, and these mechanisms of action include impaired membrane function, the generation of ROS, loss in enzyme activity, and protein dysfunction, as well as the release of toxic ions, which can be effective in eliminating drug-resistant bacteria [189,190,191]. The antimicrobial activity of these compounds is related to their particle size, stability, drug concentration, and specific surface area in contact with microorganisms [192,193]. Thus, a greater antimicrobial effect can be exerted when used in smaller sizes (e.g., nanoparticles (NPs)) because they have a larger surface area in contact with pathogens. Metallic nanoparticles are characterized by their small particle size, which can penetrate bacterial membranes and block important molecular pathways, leading to bacterial lysis [194], which shows unique antibacterial properties and could be of interest for the treatment of drug-resistant bacterial infections. Silver (Ag) has strong antibacterial potential and is an effective inhibitor of a wide range of gram-positive and gram-negative pathogens [195]. AgNPs are capable of resensitizing aminoglycoside and β-lactam antibiotics, as well as of expanding the spectrum of action of glycopeptides [195,196]. In addition, these metallic NPs include gold nanoparticles (AuNPs), zinc nanoparticles (ZnNPs), copper nanoparticles (CuNPs), and aluminum nanoparticles (AlNPs), among others [189]. Like metal nanoparticles, certain metal oxide nanoparticles have been found to have antimicrobial efficacy, including zinc oxide nanoparticles (ZnONPs), copper oxide nanoparticles (CuONPs), titanium dioxide nanoparticles (TiO_2_ NPs), magnesium oxide nanoparticles (MgONPs), and aluminum oxide nanoparticles (Al_2_O_3_NPs). Although metal or metal oxide nanoparticles have good antimicrobial effects in their own right, their application is limited by their own toxic effects, and the combined use of nanomaterials and antibiotics has proven to reduce the amount of nanomaterials, thereby reducing the toxic effects of the drugs on the cells [197] and exerting excellent synergistic antimicrobial effects. Currently, the potentially synergistic mechanisms of antibiotics combined with metal nanoparticles include the disruption of membrane structure, disruption of the electron transport chain, ROS generation, and disruption of intracellular structures (nucleic acids, proteins, and enzymes) [189,198] (Figure 6). Dove et al. [199] confirmed the synergistic effect of silver nanoparticles and aminoglycoside antibiotics against MDR *Enterococcus feacium*, with AgNPs lowering the minimal inhibitory concentration of aminoglycoside antibiotics by approximately 22-fold at a safe dose. In addition, Adeniji et al. [200] demonstrated a synergistic effect of ZnONPs in combination with vancomycin, as well as ampicillin against MDR *Enterococcus feacium*. In addition, nanoparticles are suitable for binding or carrying antibiotics, which can be used as drug carriers to improve the pharmacokinetics of antibiotics and promote the accumulation of antibiotics, thereby improving the therapeutic effect of antibiotics [201,202,203]. For example, Wang et al. [204] successfully developed a mesoporous silica nanocarrier (Ag@MSNs@LEVO) loaded with levofloxacin (LEVO) and embedded in a silver core, which can treat drug-resistant bacterial infections in vitro and in vivo. In a mouse model of acute peritonitis, treatment with Ag@MSNs@LEVO reduced the number of resistant *E. coli* GN102 by nearly three orders of magnitude.

Interestingly, in recent years, nanoenzymes, a class of mimetic enzymes that have both the unique properties of nanomaterials and catalytic functions, have received much attention in the fight against MDR pathogens. Nanoenzymes are diverse but most of them mediate catalytic reactions (mainly redox reactions) [205]. The redox-catalyzed activity of nanoenzymes generates a free radical storm that breaks the ROS balance, thus destroying the integrity of cell membranes, degrading a wide range of molecules, including nucleic acids, proteins, polysaccharides, and lipids, and destroying the morphology of bacteria, thereby killing MDR bacteria [206,207,208,209]. Yao et al. [210] reported the use of pathogen-targeting bimetallic BiPt nanozymes exhibiting dual-enzymatic activities, including peroxidase-mimic and oxidase-mimic activities, for the nanocatalytic treatment of an MDR pathogen. Further wrapping with platelet-bacterial hybrid membranes allows for a more precise and efficient clearance of carbapenem-resistant *Enterobacteriaceae* and MRSA in an osteomyelitis rat model, muscle-infected mice model, and pneumonia mice model. In addition, recent studies [211] have shown that Ag-Fe_3_O_4_@MoS_2_-magnetic NP nanocomposites loaded with DNase and vancomycin can exhibit stronger biofilm destruction and bacterial killing. Overall, these studies support the notion that the combination of antibiotics with metals and metal oxides is effective in fighting multidrug-resistant bacteria.

### 4.5. Combination Antibiotics and Phages

**Figure 7:**
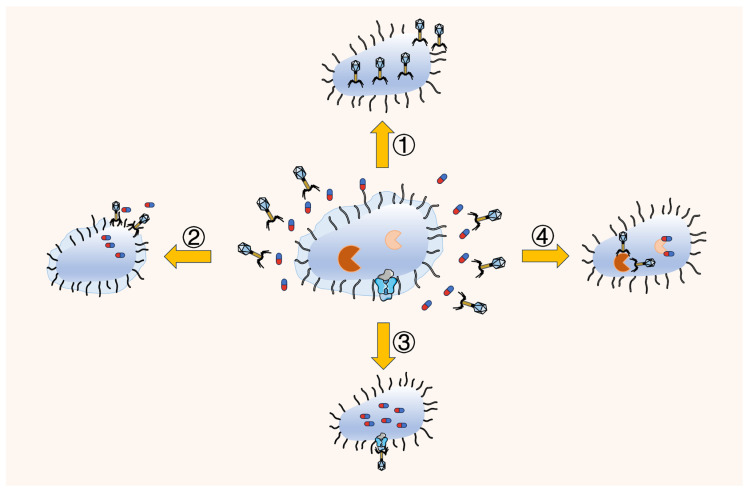
The synergistic action between phages and antibiotics: ① antibiotic-induced phage production; ② phage-induced penetration of antibiotics into biofilm; ③ phages inhibit efflux pumps to reduce efflux of intracellular antibiotics; ④ phages and antibiotics target different bacterial sites to enhance each other’s effectiveness.

Phage is a bacterial virus that widely exists in nature and can infect and kill bacteria. Compared with antibiotics, its unique advantages in the treatment of MDR bacteria have attracted widespread attention in recent years. These advantages include high host specificity [212,213], low dose requirement, low cost [214,215], high safety, anti-biofilm activity [216,217], and poor ability to produce resistance. Phage therapy alone has indeed made some outstanding advances against VRE, β-lactam-resistant *Enterobacteriaceae*, and MRSA, suggesting that phage therapy may be an alternative to antibiotics for the treatment of antibiotic-resistant bacteria [218]. However, more and more studies (Table S3) have shown that the combination of phage and antibiotics seems to have advantages over phage therapy alone [219,220,221]. Oechslin et al. [222] found a highly synergistic effect of a single dose of phage combined with ciprofloxacin in the treatment of experimental peritoneal endocarditis caused by *P. aeruginosa* through in vivo experiments in mice. The combination treatment killed >6 log CFU per gram of tissues, which is twice as many as for each of the single-agent treatments. Shlezinger et al. [223] found that the combination of vancomycin and phage EFLK1 significantly improved the efficacy of vancomycin-resistant enterococci compared with a single-drug treatment. More interestingly, Chan et al. [224] demonstrated that the selective pressure exerted by the OMKO1 phage on the MDR *P. aeruginosa* population led to the selection of an efflux pump porin OprM mutant with significantly increased antibiotic sensitivity, which, in turn, restored the antibiotic sensitivity of the resistant bacteria. In 2007, Comeau et al. [225] proposed for the first time that phage-antibiotic synergy (PAS), that is, sublethal concentrations of antibiotics, can greatly increase the yield of bacterial lytic phages. This traditional concept of PAS has been expanded with the discovery of additional mechanisms by which synergism occurs between phages and antibiotics (Figure 7). Previous studies have employed different experimental models to determine the synergistic effects of different types of phages with antibiotics, including plaque assessment, the elimination of resistant or phage bacteria, a reduction in the number of bacteria embedded in biofilms, and in vivo assessment [226]. The phage-antibiotic combination has shown possible advantages such as enhanced bacterial inhibition, significantly affecting the rate of phage adsorption and the incubation period during infection [227,228], as well as more efficient penetration into biofilms [220,229] and the reduced ability of bacteria to develop phage and/or antibiotic resistance [230]. Interestingly, when phages and antibiotics are combined, an “ordering” effect may occur so that the maximum killing effect can be achieved using phage treatment before antibiotics, and the administration time of combined therapy is optimized to enhance its efficacy [220,231]. In addition, phage infection pressure on *Vibrio cholerae* induces mutations that encode the outer membrane porin OmpU, resulting in at least a 100-fold attenuated virulence of the bacterium [232]. These findings suggest that phages combined with antibiotics have synergistic effects on host bacteria and alter the expression of bacterial virulence factors, antibiotic resistance, and growth factor activity, leading to increased antibiotic sensitivity or the inhibition of bacterial growth. Therefore, the combination of phages and antibiotics is considered to be a promising therapeutic strategy against MDR bacterial infections.

## 5. Conclusions and Future Perspectives

The combination of non-antibiotic compounds and antibiotics may be one of the main directions to address bacterial resistance, which is supported by the successful combination of amoxicillin with the β-lactamase inhibitor clavulanic acid. Based on this, over the past decade, the screening, identification, and research of new non-antibiotic and antibacterial synergies have increased day by day. In this review, we attempt to summarize the antibiotic synergistic pathways and their antimicrobial potentiates that target existing resistance mechanisms. In addition, we discuss several promising antimicrobial potentiators, including plant-derived active ingredients, metabolites, AMPs, metals and metal oxides, and phages, which can cooperate with antibiotics to overcome resistance to existing antibiotics. These antibiotics potentiate extend the lifespan of currently used antibiotics by directly or indirectly increasing their potency against resistant bacteria. In China, Chinese herbal medicine has a long history of treating diseases and abundant resources, which provide a variety of options for screening antibiotic synergies. In addition, the plant-derived active ingredients are widely sourced, have diverse structures, and have a low resistance, making them worthy of further study. Interestingly, in recent years, due to their many characteristics and advantages in combination with antibiotics, metal nanomaterials have become a desired tool to fight MDR bacteria. Remarkably, a polymeric nanoparticle, dendrimer, with its nanometric size, multivalency, biocompatibility, and structural perfection, further increases the possibilities of nanotechnology applications [233]. Dendrimers, as antibacterial agents and nanocarriers of antibacterial drugs, may have a promising prospect in combating MDR bacteria, which is worthy of attention.

However, although many non-antibiotic compounds with synergistic effects with antibiotics have been identified or discovered in vitro, only a few are also effective in animal models in vivo, and few have been approved for marketing or clinical trials. This indicates that there are still some unanswered questions, including, but not limited to, the following: (1). What is the synergy’s stability, efficacy, and safety in vivo? (2). What are the combined drugs’ pharmacokinetics, pharmacodynamics, and bioavailability distributions in vivo? (3). What are the toxicity and side effects of combination drugs? (4). Is there resistance to the compound? (5). In what dosage form and in what way can these combination drugs be delivered to the site of infection in the body to be effective? These problems need to be solved urgently.

In addition, it is necessary to accurately and efficiently screen the target compounds from the vast array of compounds. Recently, Stokes et al. [234] found potent antibiotics in more than 100 million molecules using Artificial Intelligence (AI) technology for the first time, suggesting that AI will be a powerful tool for future antibiotic potentiator screening. Therefore, in future work, it is very important to use AI methods combined with high-throughput screening platforms and molecular biology techniques to screen non-antibiotic and antibacterial synergists and study the mechanisms by which they improve the efficacy of antibiotics. However, it is still necessary to further study the mechanism of reversing bacterial resistance and explore the relationship between the reversal mechanism and drug resistance mechanisms, as well as the synergistic mechanism of synergists and antibiotics, which will effectively accelerate the development of non-antibiotic synergists. In summary, the combination therapy of antibiotic and non-antibiotic compounds is promising and has great commercial potential; however, there are also many problems to be solved.

## Figures and Tables

**Table 1:** Representative synergistic combinations between plant-derived active ingredients and antibiotics against drug-resistant bacteria.

Phytochemical	Combination with Antibiotic	Antibiotic-Resistance Bacteria	Mechanism of Action	Reference
Flavonoids				
Baicalein	Doxycycline	MDR gram-negative pathogens	Inhibited multidrug efflux pumps and biofilm formation and disrupted the membrane	[102]
Catechol-type flavonoids-7,8-dihydroxyflavone, myricetin, and luteolin	Colistin	Colistin-resistant bacteria	Disrupted iron homeostasis and interfered with pmrA/pmrB system	[123]
Hibifolin	Cefotaxime	MRSA	Inhibited Sortase A (SrtA) activity	[131]
Hinokiflavone	Vancomycin	MRSA	Inhibited Caseinolytic protease P (ClpP) activity	[132]
Scutellarin	Vancomycin	MRSA	Dual inhibition of SrtA and ClpP	[133]
Quercetin	Meropenem	Carbapenem-resistant gram-negative bacteria	Inhibited carbapenemase and efflux pumps	[134]
Baicalein	Colistin	Colistin-resistant *Salmonella*	---	[135]
α-mangostin and isobavachalcone	Colistin	Colistin-resistant gram-negative pathogens	Dissipation of PMF and metabolic perturbations	[136]
Quercetin	Tetracycline	MDR *E. coli* carrying *mcr-1* and *tet* resistance genes	Disrupted the bacterial cell envelope resulting in increased permeability and cell lysis	[137]
Rhamnetin 3-O-(6″-galloyl)-β-D-glucopyranoside, quercetin 3-O-(6″-galloyl)-β-D-glucopyranoside	Methicillin	MRSA	Remodeling metabolism	[138]
Naringenin	Colistin	MDR *K. pneumoniae*	Inhibited mcr gene activity and repression of two-component system	[139]
Phloretin	Colistin	*E. coli* ZJ478 or *Salmonella* sp. stain HYM2	---	[140]
Isoliquiritin	Isoliquiritin	NDM-1-positive *Enterobacteriaceae*	Inhibited NDM-1 enzyme activity	[74]
Kaempferol	Colistin	Colistin-resistant gram-negative bacteria	Reduced the number of bacteria in the biofilm	[141]
Ceragenin	Ceftazidime, Levofloxacin, Co-Trimoxazole, and Colistin	*Stenotrophomonas maltophilia*	---	[142]
Alkaloids				
Tetrandrine	Colistin	MCR-positive *salmonella*	Undermined PMF and efflux pumps; inhibited the expression of MCR-1	[143]
Tetrandrine	Colistin	MCR-positive *E. coli*	Inhibited MCR-1 from binding to its substrates	[144]
Budmunchiamines	Chloramphenicol	Chloramphenicol-resistant bacteria	Inhibit acrB efflux pump	[145]
Berberine hydrochloride	Tigecycline, Sulbactam, Meropenem, and Ciprofloxacin	MDR *A. baumannii*	Boosted AdeB gene expression and bound to the adeb transporter protein	[146]
1,4-naphthoquinone	Imipenem, Cefuroxime, and Cefotaxime	MRSA	---	[147]
Chanoclavine	Tetracycline	MDR *E. coli*	Inhibited ATPase-dependent efflux pumps	[148]
Berberine chloride	Vancomycin	*Clostridioides difficile*	---	[149]
Terpenes				
Pogostone	Colistin	MCR-1-positive bacteria	Inhibited the binding of MCR-1 to substrates	[150]
Carvacrol and Thymol	Norfloxacin	*S. aureus* SA-1199B	Inhibit NorA	[151]
Celastrol	Vancomycin	Vancomycin-resistant enterococci	Inhibited bacterial cell-division protein FtsZ	[152]
Cryptotanshinone	Fosfomycin	Fosfomycin-resistant *S. aureus*	---	[153]
Monoterpene	Tetracycline, Erythromycin	*S. aureus*	Inhibited efflux pumps	[154]
Dihydroartemisinin	Colistin	MCR-1-positive, gram-negative bacteria	Inhibited mcr-1 activity; disrupted energy metabolism	[155]
Corosolic Acid	Carbapenems	KPC-2-positive strain, *E. coli* BL21(DE3) (pet28a-KPC-2)	Inhibited β-lactamase KPC-2 activity	[156]
Isoalantolactone	Penicillin G	β-Lactamase-positive *S. aureus*	Inactivated β-lactamase	[157]
Cannabidiol	Polymyxin	MDR polymyxin-resistant gram-negative bacteria	Disrupted the DNA and RNA biosynthetic pathways	[158]
Phenols				
Proanthocyanidins	β-Lactam antibiotics (cefotaxime; meropenem)	β-lactam-resistant Enterobacteriaceae and staphylococci (*E. coli, Klebsiella,* MRSA)	Inhibited β-lactamase activity	[159]
Salicylate, curcumin	Colistin	Carbapenem resistant Enterobacteriaceae	Inhibited MraR expression and the efflux pump	[160]
Honokiol	Colistin	MCR-1-positive Enterobacteriaceae	Reduced MCR-1 activity	[161]
Resveratrol	Colistin	Colistin-resistant *P. aeruginosa*	Increased membrane permeability	[162]
Thymol	Colistin	Gram-negative bacteria, including nonfermenting bacteria and Enterobacteriaceae.	Damaged bacterial outer membrane and increased permeability	[163]
Other				
Hypericin	β-Lactam antibiotics (oxacillin, cefazolin and nafcillin)	β-lactam-resistant MRSA	Inhibited SarA expression and reduced biofilm formation	[164]
Verbascoside	Vancomycin, Ceftazidime	MDR *S. aureus* SA-596, MDR *P. aeruginosa* PA-69	Cell membrane dysfunction; biofilm eradication	[165]
6-gingerol analog	Tobramycin	*P. aeruginosa*	Inhibited biofilm formation and RhlR inactivation	[166]
Plumbagin	Tet(X3)/tet(X4)-positive bacteria	Tet(X3)/tet(X4)-positive bacteria	Inhibited the activity of monooxygenases; increased oxidative stress and metabolism	[167]
Nordihydroguaiaretic acid	Colistin	MCR-1-positive *E. coli* ZJ487	Directly inhibited MCR-1 activity and injured the bacterial cell membrane	[168]

## Supplementary Materials

The supporting information can be downloaded at: https://www.mdpi.com/article/10.3390/ijms242015493/s1, References [235,236,237,238,239,240,241,242,243,244,245,246,247,248,249,250,251,252,253,254,255,256,257,258,259,260,261,262,263,264,265,266,267,268,269,270,271,272,273,274,275,276,277] are cited in the Supplementary Materials.

## References

[1] Antimicrobial Resistance Collaborators (2022). Global burden of bacterial antimicrobial resistance in 2019: A systematic analysis. Lancet.

[2] O’Neill J. (2016). Tackling Drug-Resistant Infections Globally: Final Report and Recommendations.

[3] Liu Y.Y., Wang Y., Walsh T.R., Yi L.X., Zhang R., Spencer J., Doi Y., Tian G., Dong B., Huang X. (2016). Emergence of plasmid-mediated colistin resistance mechanism MCR-1 in animals and human beings in China: A microbiological and molecular biological study. Lancet Infect. Dis..

[4] Andrei S., Droc G., Stefan G. (2019). FDA approved antibacterial drugs: 2018–2019. Discoveries.

[5] Cooper M.A., Shlaes D. (2011). Fix the antibiotics pipeline. Nature.

[6] Walsh C. (2000). Molecular mechanisms that confer antibacterial drug resistance. Nature.

[7] Tyers M., Wright G.D. (2019). Drug combinations: A strategy to extend the life of antibiotics in the 21st century. Nat. Rev. Microbiol..

[8] Worthington R.J., Melander C. (2013). Combination approaches to combat multidrug-resistant bacteria. Trends Biotechnol..

[9] Salter A.J. (1971). Trimethoprim-sulphamethoxazole. Drugs.

[10] Liu Y., Tong Z., Shi J., Li R., Upton M., Wang Z. (2021). Drug repurposing for next-generation combination therapies against multidrug-resistant bacteria. Theranostics.

[11] Ninane G., Joly J., Kraytman M., Piot P. (1978). Bronchopulmonary infection due to beta-lactamase-producing Branhamella catarrhalis treated with amoxycillin/clavulanic-acid. Lancet.

[12] Grant T.A., Lopez-Perez M., Haro-Moreno J.M., Almagro-Moreno S. (2023). Allelic diversity uncovers protein domains contributing to the emergence of antimicrobial resistance. PLoS Genet..

[13] Huo W., Busch L.M., Hernandez-Bird J., Hamami E., Marshall C.W., Geisinger E., Cooper V.S., van Opijnen T., Rosch J.W., Isberg R.R. (2022). Immunosuppression broadens evolutionary pathways to drug resistance and treatment failure during *Acinetobacter baumannii* pneumonia in mice. Nat. Microbiol..

[14] D’Costa V.M., McGrann K.M., Hughes D.W., Wright G.D. (2006). Sampling the antibiotic resistome. Science.

[15] Pawlowski A.C., Wang W., Koteva K., Barton H.A., McArthur A.G., Wright G.D. (2016). A diverse intrinsic antibiotic resistome from a cave bacterium. Nat. Commun..

[16] De Pascale G., Wright G.D. (2010). Antibiotic resistance by enzyme inactivation: From mechanisms to solutions. Chembiochem.

[17] Tooke C.L., Hinchliffe P., Bragginton E.C., Colenso C.K., Hirvonen V.H.A., Takebayashi Y., Spencer J. (2019). beta-Lactamases and beta-Lactamase Inhibitors in the 21st Century. J. Mol. Biol..

[18] Yang Y., Yan Y.H., Schofield C.J., McNally A., Zong Z., Li G.B. (2023). Metallo-beta-lactamase-mediated antimicrobial resistance and progress in inhibitor discovery. Trends Microbiol..

[19] Arer V., Kar D. (2022). Biochemical exploration of beta-lactamase inhibitors. Front. Genet..

[20] Mojica M.F., Rossi M.A., Vila A.J., Bonomo R.A. (2022). The urgent need for metallo-beta-lactamase inhibitors: An unattended global threat. Lancet Infect. Dis..

[21] Kumarasamy K.K., Toleman M.A., Walsh T.R., Bagaria J., Butt F., Balakrishnan R., Chaudhary U., Doumith M., Giske C.G., Irfan S. (2010). Emergence of a new antibiotic resistance mechanism in India, Pakistan, and the UK: A molecular, biological, and epidemiological study. Lancet Infect. Dis..

[22] Johnson A.P., Woodford N. (2013). Global spread of antibiotic resistance: The example of New Delhi metallo-beta-lactamase (NDM)-mediated carbapenem resistance. J. Med. Microbiol..

[23] Khan A.U., Maryam L., Zarrilli R. (2017). Structure, Genetics and Worldwide Spread of New Delhi Metallo-beta-lactamase (NDM): A threat to public health. BMC Microbiol..

[24] Pitout J.D.D., Peirano G., Kock M.M., Strydom K.A., Matsumura Y. (2019). The Global Ascendency of OXA-48-Type Carbapenemases. Clin. Microbiol. Rev..

[25] Potter R.F., D’Souza A.W., Dantas G. (2016). The rapid spread of carbapenem-resistant Enterobacteriaceae. Drug Resist. Updates.

[26] Wright G.D. (2005). Bacterial resistance to antibiotics: Enzymatic degradation and modification. Adv. Drug Deliv. Rev..

[27] Azucena E., Mobashery S. (2001). Aminoglycoside-modifying enzymes: Mechanisms of catalytic processes and inhibition. Drug Resist. Updates.

[28] Ramirez M.S., Tolmasky M.E. (2010). Aminoglycoside modifying enzymes. Drug Resist. Updates.

[29] Wright G.D. (1999). Aminoglycoside-modifying enzymes. Curr. Opin. Microbiol..

[30] Bordeleau E., Stogios P.J., Evdokimova E., Koteva K., Savchenko A., Wright G.D. (2021). ApmA Is a Unique Aminoglycoside Antibiotic Acetyltransferase That Inactivates Apramycin. mBio.

[31] Darby E.M., Trampari E., Siasat P., Gaya M.S., Alav I., Webber M.A., Blair J.M.A. (2023). Molecular mechanisms of antibiotic resistance revisited. Nat. Rev. Microbiol..

[32] Luthje P., von Kockritz-Blickwede M., Schwarz S. (2007). Identification and characterization of nine novel types of small staphylococcal plasmids carrying the lincosamide nucleotidyltransferase gene lnu(A). J. Antimicrob. Chemother..

[33] Boumghar-Bourtchai L., Mariani-Kurkdjian P., Bingen E., Filliol I., Dhalluin A., Ifrane S.A., Weill F.X., Leclercq R. (2008). Macrolide-resistant Shigella sonnei. Emerg. Infect. Dis..

[34] Schaenzer A.J., Wright G.D. (2020). Antibiotic Resistance by Enzymatic Modification of Antibiotic Targets. Trends Mol. Med..

[35] Lambert P.A. (2005). Bacterial resistance to antibiotics: Modified target sites. Adv. Drug Deliv. Rev..

[36] Smith W.P.J., Wucher B.R., Nadell C.D., Foster K.R. (2023). Bacterial defences: Mechanisms, evolution and antimicrobial resistance. Nat. Rev. Microbiol..

[37] Floss H.G., Yu T.W. (2005). Rifamycin-mode of action, resistance, and biosynthesis. Chem. Rev..

[38] Jin D.J., Gross C.A. (1988). Mapping and sequencing of mutations in the *Escherichia coli* rpoB gene that lead to rifampicin resistance. J. Mol. Biol..

[39] Leclercq R. (2002). Mechanisms of resistance to macrolides and lincosamides: Nature of the resistance elements and their clinical implications. Clin. Infect. Dis..

[40] Weisblum B. (1995). Erythromycin resistance by ribosome modification. Antimicrob. Agents Chemother..

[41] Toh S.M., Xiong L., Arias C.A., Villegas M.V., Lolans K., Quinn J., Mankin A.S. (2007). Acquisition of a natural resistance gene renders a clinical strain of methicillin-resistant *Staphylococcus aureus* resistant to the synthetic antibiotic linezolid. Mol. Microbiol..

[42] Schwarz S., Werckenthin C., Kehrenberg C. (2000). Identification of a plasmid-borne chloramphenicol-florfenicol resistance gene in Staphylococcus sciuri. Antimicrob. Agents Chemother..

[43] Larsen J., Raisen C.L., Ba X., Sadgrove N.J., Padilla-Gonzalez G.F., Simmonds M.S.J., Loncaric I., Kerschner H., Apfalter P., Hartl R. (2022). Emergence of methicillin resistance predates the clinical use of antibiotics. Nature.

[44] Stapleton P.D., Taylor P.W. (2002). Methicillin resistance in *Staphylococcus aureus*: Mechanisms and modulation. Sci. Prog..

[45] Caveney N.A., Caballero G., Voedts H., Niciforovic A., Worrall L.J., Vuckovic M., Fonvielle M., Hugonnet J.E., Arthur M., Strynadka N.C.J. (2019). Structural insight into YcbB-mediated beta-lactam resistance in *Escherichia coli*. Nat. Commun..

[46] Fernandez L., Hancock R.E. (2012). Adaptive and mutational resistance: Role of porins and efflux pumps in drug resistance. Clin. Microbiol. Rev..

[47] Tsai Y.K., Fung C.P., Lin J.C., Chen J.H., Chang F.Y., Chen T.L., Siu L.K. (2011). *Klebsiella pneumoniae* outer membrane porins OmpK35 and OmpK36 play roles in both antimicrobial resistance and virulence. Antimicrob. Agents Chemother..

[48] Hasdemir U.O., Chevalier J., Nordmann P., Pages J.M. (2004). Detection and prevalence of active drug efflux mechanism in various multidrug-resistant *Klebsiella pneumoniae* strains from Turkey. J. Clin. Microbiol..

[49] Webber M.A., Piddock L.J. (2003). The importance of efflux pumps in bacterial antibiotic resistance. J. Antimicrob. Chemother..

[50] Henderson P.J.F., Maher C., Elbourne L.D.H., Eijkelkamp B.A., Paulsen I.T., Hassan K.A. (2021). Physiological Functions of Bacterial “Multidrug” Efflux Pumps. Chem. Rev..

[51] Si Z., Pethe K., Chan-Park M.B. (2023). Chemical Basis of Combination Therapy to Combat Antibiotic Resistance. JACS Au.

[52] Symmons M.F., Bokma E., Koronakis E., Hughes C., Koronakis V. (2009). The assembled structure of a complete tripartite bacterial multidrug efflux pump. Proc. Natl. Acad. Sci. USA.

[53] Jang S. (2023). AcrAB-TolC, a major efflux pump in Gram negative bacteria: Toward understanding its operation mechanism. BMB Rep..

[54] Aeschlimann J.R. (2003). The role of multidrug efflux pumps in the antibiotic resistance of *Pseudomonas aeruginosa* and other gram-negative bacteria. Insights from the Society of Infectious Diseases Pharmacists. Pharmacotherapy.

[55] Poole K., Srikumar R. (2001). Multidrug efflux in *Pseudomonas aeruginosa*: Components, mechanisms and clinical significance. Curr. Top. Med. Chem..

[56] Schrader S.M., Vaubourgeix J., Nathan C. (2020). Biology of antimicrobial resistance and approaches to combat it. Sci. Transl. Med..

[57] Yang J.H., Wright S.N., Hamblin M., McCloskey D., Alcantar M.A., Schrubbers L., Lopatkin A.J., Satish S., Nili A., Palsson B.O. (2019). A White-Box Machine Learning Approach for Revealing Antibiotic Mechanisms of Action. Cell.

[58] Bhargava P., Collins J.J. (2015). Boosting bacterial metabolism to combat antibiotic resistance. Cell Metab..

[59] Stokes J.M., Lopatkin A.J., Lobritz M.A., Collins J.J. (2019). Bacterial Metabolism and Antibiotic Efficacy. Cell Metab..

[60] Lopatkin A.J., Bening S.C., Manson A.L., Stokes J.M., Kohanski M.A., Badran A.H., Earl A.M., Cheney N.J., Yang J.H., Collins J.J. (2021). Clinically relevant mutations in core metabolic genes confer antibiotic resistance. Science.

[61] Lobritz M.A., Belenky P., Porter C.B., Gutierrez A., Yang J.H., Schwarz E.G., Dwyer D.J., Khalil A.S., Collins J.J. (2015). Antibiotic efficacy is linked to bacterial cellular respiration. Proc. Natl. Acad. Sci. USA.

[62] Amato S.M., Orman M.A., Brynildsen M.P. (2013). Metabolic control of persister formation in *Escherichia coli*. Mol. Cell..

[63] Zampieri M., Zimmermann M., Claassen M., Sauer U. (2017). Nontargeted Metabolomics Reveals the Multilevel Response to Antibiotic Perturbations. Cell Rep..

[64] Vincent I.M., Ehmann D.E., Mills S.D., Perros M., Barrett M.P. (2016). Untargeted Metabolomics To Ascertain Antibiotic Modes of Action. Antimicrob. Agents Chemother..

[65] Peng B., Su Y.B., Li H., Han Y., Guo C., Tian Y.M., Peng X.X. (2015). Exogenous alanine and/or glucose plus kanamycin kills antibiotic-resistant bacteria. Cell Metab..

[66] Alonso A., Morales G., Escalante R., Campanario E., Sastre L., Martinez J.L. (2004). Overexpression of the multidrug efflux pump SmeDEF impairs *Stenotrophomonas maltophilia* physiology. J. Antimicrob. Chemother..

[67] Stickland H.G., Davenport P.W., Lilley K.S., Griffin J.L., Welch M. (2010). Mutation of nfxB causes global changes in the physiology and metabolism of *Pseudomonas aeruginosa*. J. Proteome Res..

[68] Neu H.C., Fu K.P. (1978). Clavulanic acid, a novel inhibitor of beta-lactamases. Antimicrob. Agents Chemother..

[69] Reading C., Cole M. (1977). Clavulanic acid: A beta-lactamase-inhiting beta-lactam from *Streptomyces clavuligerus*. Antimicrob. Agents Chemother..

[70] (2015). Ceftazidime/Avibactam (Avycaz)—A new intraveneous antibiotic. Med. Lett. Drugs Ther..

[71] Cho J.C., Zmarlicka M.T., Shaeer K.M., Pardo J. (2018). Meropenem/Vaborbactam, the First Carbapenem/beta-Lactamase Inhibitor Combination. Ann. Pharmacother..

[72] Durand-Reville T.F., Guler S., Comita-Prevoir J., Chen B., Bifulco N., Huynh H., Lahiri S., Shapiro A.B., McLeod S.M., Carter N.M. (2017). ETX2514 is a broad-spectrum beta-lactamase inhibitor for the treatment of drug-resistant Gram-negative bacteria including *Acinetobacter baumannii*. Nat. Microbiol..

[73] King A.M., Reid-Yu S.A., Wang W., King D.T., De Pascale G., Strynadka N.C., Walsh T.R., Coombes B.K., Wright G.D. (2014). Aspergillomarasmine A overcomes metallo-beta-lactamase antibiotic resistance. Nature.

[74] Wang Y., Sun X., Kong F., Xia L., Deng X., Wang D., Wang J. (2020). Specific NDM-1 Inhibitor of Isoliquiritin Enhances the Activity of Meropenem against NDM-1-positive Enterobacteriaceae in vitro. Int. J. Environ. Res. Public Health.

[75] Wachino J.I., Jin W., Kimura K., Kurosaki H., Sato A., Arakawa Y. (2020). Sulfamoyl Heteroarylcarboxylic Acids as Promising Metallo-beta-Lactamase Inhibitors for Controlling Bacterial Carbapenem Resistance. mBio.

[76] Guo Y., Yang Y., Xu X., Li L., Zhou Y., Jia G., Wei L., Yu Q., Wang J. (2022). Metallo-beta-lactamases inhibitor fisetin attenuates meropenem resistance in NDM-1-producing *Escherichia coli*. Eur. J. Med. Chem..

[77] Zalacain M., Lozano C., Llanos A., Sprynski N., Valmont T., De Piano C., Davies D., Leiris S., Sable C., Ledoux A. (2021). Novel Specific Metallo-beta-Lactamase Inhibitor ANT2681 Restores Meropenem Activity to Clinically Effective Levels against NDM-Positive Enterobacterales. Antimicrob. Agents Chemother..

[78] Zhu L., Liu R., Liu T., Zou X., Xu Z., Guan H. (2019). A novel strategy to screen inhibitors of multiple aminoglycoside-modifying enzymes with ultra-high performance liquid chromatography-quadrupole-time-of-flight mass spectrometry. J. Pharm. Biomed. Anal..

[79] Boehr D.D., Draker K.A., Koteva K., Bains M., Hancock R.E., Wright G.D. (2003). Broad-spectrum peptide inhibitors of aminoglycoside antibiotic resistance enzymes. Chem. Biol..

[80] Ple C., Tam H.K., Vieira Da Cruz A., Compagne N., Jimenez-Castellanos J.C., Muller R.T., Pradel E., Foong W.E., Malloci G., Ballee A. (2022). Pyridylpiperazine-based allosteric inhibitors of RND-type multidrug efflux pumps. Nat. Commun..

[81] Yang B., Tong Z., Shi J., Wang Z., Liu Y. (2023). Bacterial proton motive force as an unprecedented target to control antimicrobial resistance. Med. Res. Rev..

[82] Goldberg K., Sarig H., Zaknoon F., Epand R.F., Epand R.M., Mor A. (2013). Sensitization of gram-negative bacteria by targeting the membrane potential. FASEB J..

[83] Compagne N., Vieira Da Cruz A., Muller R.T., Hartkoorn R.C., Flipo M., Pos K.M. (2023). Update on the Discovery of Efflux Pump Inhibitors against Critical Priority Gram-Negative Bacteria. Antibiotics.

[84] Jamshidi S., Sutton J.M., Rahman K.M. (2017). Computational Study Reveals the Molecular Mechanism of the Interaction between the Efflux Inhibitor PAbetaN and the AdeB Transporter from *Acinetobacter baumannii*. ACS Omega.

[85] Kinana A.D., Vargiu A.V., May T., Nikaido H. (2016). Aminoacyl beta-naphthylamides as substrates and modulators of AcrB multidrug efflux pump. Proc. Natl. Acad. Sci. USA.

[86] Blanchard C., Barnett P., Perlmutter J., Dunman P.M. (2014). Identification of *Acinetobacter baumannii* serum-associated antibiotic efflux pump inhibitors. Antimicrob. Agents Chemother..

[87] Stone L.K., Baym M., Lieberman T.D., Chait R., Clardy J., Kishony R. (2016). Compounds that select against the tetracycline-resistance efflux pump. Nat. Chem. Biol..

[88] Sjuts H., Vargiu A.V., Kwasny S.M., Nguyen S.T., Kim H.S., Ding X., Ornik A.R., Ruggerone P., Bowlin T.L., Nikaido H. (2016). Molecular basis for inhibition of AcrB multidrug efflux pump by novel and powerful pyranopyridine derivatives. Proc. Natl. Acad. Sci. USA.

[89] Domalaon R., Idowu T., Zhanel G.G., Schweizer F. (2018). Antibiotic Hybrids: The Next Generation of Agents and Adjuvants against Gram-Negative Pathogens?. Clin. Microbiol. Rev..

[90] Stokes J.M., MacNair C.R., Ilyas B., French S., Cote J.P., Bouwman C., Farha M.A., Sieron A.O., Whitfield C., Coombes B.K. (2017). Pentamidine sensitizes Gram-negative pathogens to antibiotics and overcomes acquired colistin resistance. Nat. Microbiol..

[91] Lazzaro B.P., Zasloff M., Rolff J. (2020). Antimicrobial peptides: Application informed by evolution. Science.

[92] Ma B., Fang C., Lu L., Wang M., Xue X., Zhou Y., Li M., Hu Y., Luo X., Hou Z. (2019). The antimicrobial peptide thanatin disrupts the bacterial outer membrane and inactivates the NDM-1 metallo-beta-lactamase. Nat. Commun..

[93] Andrade F.F., Silva D., Rodrigues A., Pina-Vaz C. (2020). Colistin Update on Its Mechanism of Action and Resistance, Present and Future Challenges. Microorganisms.

[94] Si Z., Lim H.W., Tay M.Y.F., Du Y., Ruan L., Qiu H., Zamudio-Vazquez R., Reghu S., Chen Y., Tiong W.S. (2020). A Glycosylated Cationic Block Poly(beta-peptide) Reverses Intrinsic Antibiotic Resistance in All ESKAPE Gram-Negative Bacteria. Angew. Chem. Int. Ed. Engl..

[95] Hwang H.J., Li D.D., Lee J., Kang M.K., Moon H.R., Lee J.H. (2023). Compounds That Have an Anti-Biofilm Effect against Common Bacteria at Very Low Concentrations and Their Antibiotic Combination Effect. Antibiotics.

[96] Roy R., Tiwari M., Donelli G., Tiwari V. (2018). Strategies for combating bacterial biofilms: A focus on anti-biofilm agents and their mechanisms of action. Virulence.

[97] Chung P.Y., Khanum R. (2017). Antimicrobial peptides as potential anti-biofilm agents against multidrug-resistant bacteria. J. Microbiol. Immunol. Infect..

[98] Mukherjee S., Bassler B.L. (2019). Bacterial quorum sensing in complex and dynamically changing environments. Nat. Rev. Microbiol..

[99] Hengge R. (2009). Principles of c-di-GMP signalling in bacteria. Nat. Rev. Microbiol..

[100] Chen J., Lu Y., Ye F., Zhang H., Zhou Y., Li J., Wu Q., Xu X., Wu Q., Wei B. (2022). A Small-Molecule Inhibitor of the Anthranilyl-CoA Synthetase PqsA for the Treatment of Multidrug-Resistant *Pseudomonas aeruginosa*. Microbiol. Spectr..

[101] Zhang D., Yin F., Qin Q., Qiao L. (2023). Molecular responses during bacterial filamentation reveal inhibition methods of drug-resistant bacteria. Proc. Natl. Acad. Sci. USA.

[102] Wang Y., Su J., Zhou Z., Yang J., Liu W., Zhang Y., Zhang P., Guo T., Li G. (2023). Baicalein Resensitizes Multidrug-Resistant Gram-Negative Pathogens to Doxycycline. Microbiol. Spectr..

[103] Allison K.R., Brynildsen M.P., Collins J.J. (2011). Metabolite-enabled eradication of bacterial persisters by aminoglycosides. Nature.

[104] Van Acker H., Coenye T. (2017). The Role of Reactive Oxygen Species in Antibiotic-Mediated Killing of Bacteria. Trends Microbiol..

[105] Keren I., Wu Y., Inocencio J., Mulcahy L.R., Lewis K. (2013). Killing by bactericidal antibiotics does not depend on reactive oxygen species. Science.

[106] Brynildsen M.P., Winkler J.A., Spina C.S., MacDonald I.C., Collins J.J. (2013). Potentiating antibacterial activity by predictably enhancing endogenous microbial ROS production. Nat. Biotechnol..

[107] Foti J.J., Devadoss B., Winkler J.A., Collins J.J., Walker G.C. (2012). Oxidation of the guanine nucleotide pool underlies cell death by bactericidal antibiotics. Science.

[108] Kohanski M.A., Dwyer D.J., Hayete B., Lawrence C.A., Collins J.J. (2007). A common mechanism of cellular death induced by bactericidal antibiotics. Cell.

[109] Zhao X., Drlica K. (2014). Reactive oxygen species and the bacterial response to lethal stress. Curr. Opin. Microbiol..

[110] Wu T., Wang X., Dong Y., Xing C., Chen X., Li L., Dong C., Li Y. (2022). Effects of l-Serine on Macrolide Resistance in *Streptococcus suis*. Microbiol. Spectr..

[111] Liu Y., Li R., Xiao X., Wang Z. (2019). Bacterial metabolism-inspired molecules to modulate antibiotic efficacy. J. Antimicrob. Chemother..

[112] Liu Y., Yang K., Jia Y., Shi J., Tong Z., Wang Z. (2020). Cysteine Potentiates Bactericidal Antibiotics Activity Against Gram-Negative Bacterial Persisters. Infect. Drug Resist..

[113] Liu Y., Yang K., Jia Y., Shi J., Tong Z., Wang Z. (2021). Thymine Sensitizes Gram-Negative Pathogens to Antibiotic Killing. Front. Microbiol..

[114] Su Y.B., Peng B., Li H., Cheng Z.X., Zhang T.T., Zhu J.X., Li D., Li M.Y., Ye J.Z., Du C.C. (2018). Pyruvate cycle increases aminoglycoside efficacy and provides respiratory energy in bacteria. Proc. Natl. Acad. Sci. USA.

[115] Zhao X.L., Chen Z.G., Yang T.C., Jiang M., Wang J., Cheng Z.X., Yang M.J., Zhu J.X., Zhang T.T., Li H. (2021). Glutamine promotes antibiotic uptake to kill multidrug-resistant uropathogenic bacteria. Sci. Transl. Med..

[116] Chen X.H., Liu S.R., Peng B., Li D., Cheng Z.X., Zhu J.X., Zhang S., Peng Y.M., Li H., Zhang T.T. (2017). Exogenous l-Valine Promotes Phagocytosis to Kill Multidrug-Resistant Bacterial Pathogens. Front. Immunol..

[117] Jiang M., Yang L., Chen Z.G., Lai S.S., Zheng J., Peng B. (2020). Exogenous maltose enhances Zebrafish immunity to levofloxacin-resistant *Vibrio alginolyticus*. Microb. Biotechnol..

[118] Papareddy P., Kalle M., Sorensen O.E., Malmsten M., Morgelin M., Schmidtchen A. (2013). The TFPI-2 derived peptide EDC34 improves outcome of gram-negative sepsis. PLoS Pathog..

[119] Deepika M.S., Thangam R., Vijayakumar T.S., Sasirekha R., Vimala R.T.V., Sivasubramanian S., Arun S., Babu M.D., Thirumurugan R. (2019). Antibacterial synergy between rutin and florfenicol enhances therapeutic spectrum against drug resistant *Aeromonas hydrophila*. Microb. Pathog..

[120] Simoes M., Bennett R.N., Rosa E.A. (2009). Understanding antimicrobial activities of phytochemicals against multidrug resistant bacteria and biofilms. Nat. Prod. Rep..

[121] Bao M., Zhang L., Liu B., Li L., Zhang Y., Zhao H., Ji X., Chen Q., Hu M., Bai J. (2020). Synergistic effects of anti-MRSA herbal extracts combined with antibiotics. Future Microbiol..

[122] Catteau L., Reichmann N.T., Olson J., Pinho M.G., Nizet V., Van Bambeke F., Quetin-Leclercq J. (2017). Synergy between Ursolic and Oleanolic Acids from Vitellaria paradoxa Leaf Extract and beta-Lactams against Methicillin-Resistant *Staphylococcus aureus*: In Vitro and In Vivo Activity and Underlying Mechanisms. Molecules.

[123] Zhong Z.X., Zhou S., Liang Y.J., Wei Y.Y., Li Y., Long T.F., He Q., Li M.Y., Zhou Y.F., Yu Y. (2023). Natural flavonoids disrupt bacterial iron homeostasis to potentiate colistin efficacy. Sci. Adv..

[124] Owen L., Laird K. (2018). Synchronous application of antibiotics and essential oils: Dual mechanisms of action as a potential solution to antibiotic resistance. Crit. Rev. Microbiol..

[125] Perricone M., Arace E., Corbo M.R., Sinigaglia M., Bevilacqua A. (2015). Bioactivity of essential oils: A review on their interaction with food components. Front. Microbiol..

[126] Aelenei P., Miron A., Trifan A., Bujor A., Gille E., Aprotosoaie A.C. (2016). Essential Oils and Their Components as Modulators of Antibiotic Activity against Gram-Negative Bacteria. Medicines.

[127] Langeveld W.T., Veldhuizen E.J., Burt S.A. (2014). Synergy between essential oil components and antibiotics: A review. Crit. Rev. Microbiol..

[128] Subramani R., Narayanasamy M., Feussner K.D. (2017). Plant-derived antimicrobials to fight against multi-drug-resistant human pathogens. 3 Biotech.

[129] Rai M., Paralikar P., Jogee P., Agarkar G., Ingle A.P., Derita M., Zacchino S. (2017). Synergistic antimicrobial potential of essential oils in combination with nanoparticles: Emerging trends and future perspectives. Int. J. Pharm..

[130] Trifan A., Luca S.V., Greige-Gerges H., Miron A., Gille E., Aprotosoaie A.C. (2020). Recent advances in tackling microbial multidrug resistance with essential oils: Combinatorial and nano-based strategies. Crit. Rev. Microbiol..

[131] Song W., Wang L., Zhao Y., Lanzi G., Wang X., Zhang C., Guan J., Wang W., Guo X., Meng Y. (2022). Hibifolin, a Natural Sortase A Inhibitor, Attenuates the Pathogenicity of *Staphylococcus aureus* and Enhances the Antibacterial Activity of Cefotaxime. Microbiol. Spectr..

[132] Kong X., Wang B., Chen X., Wang L., Wang X., Hou J., Wei L., Sui L., Zhang C., Guan J. (2022). Hinokiflavone Attenuates the Virulence of Methicillin-Resistant *Staphylococcus aureus* by Targeting Caseinolytic Protease P. Antimicrob. Agents Chemother..

[133] Wang X., Wei L., Wang L., Chen X., Kong X., Luan Y., Guan J., Guo X., Shi Y., Wang T. (2022). Scutellarin potentiates vancomycin against lethal pneumonia caused by methicillin-resistant *Staphylococcus aureus* through dual inhibition of sortase A and caseinolytic peptidase P. Biochem. Pharmacol..

[134] Pal A., Tripathi A. (2020). Quercetin inhibits carbapenemase and efflux pump activities among carbapenem-resistant Gram-negative bacteria. APMIS.

[135] Cui X.D., Zhang J.K., Sun Y.W., Yan F.B., Zhao J.F., He D.D., Pan Y.S., Yuan L., Zhai Y.J., Hu G.Z. (2023). Synergistic antibacterial activity of baicalin and EDTA in combination with colistin against colistin-resistant Salmonella. Poult. Sci..

[136] Song M., Liu Y., Li T., Liu X., Hao Z., Ding S., Panichayupakaranant P., Zhu K., Shen J. (2021). Plant Natural Flavonoids Against Multidrug Resistant Pathogens. Adv. Sci..

[137] Qu S., Dai C., Shen Z., Tang Q., Wang H., Zhai B., Zhao L., Hao Z. (2019). Mechanism of Synergy Between Tetracycline and Quercetin Against Antibiotic Resistant *Escherichia coli*. Front. Microbiol..

[138] Yu J.S., Kim J.H., Rashan L., Kim I., Lee W., Kim K.H. (2021). Potential Antimicrobial Activity of Galloyl-Flavonoid Glycosides From Woodfordia uniflora Against Methicillin-Resistant *Staphylococcus aureus*. Front. Microbiol..

[139] Sheng Q., Hou X., Wang Y., Wang N., Deng X., Wen Z., Li D., Li L., Zhou Y., Wang J. (2022). Naringenin Microsphere as a Novel Adjuvant Reverses Colistin Resistance via Various Strategies against Multidrug-Resistant *Klebsiella pneumoniae* Infection. J. Agric. Food Chem..

[140] Du R., Lv Q., Hu W., Hou X., Zhou Y., Deng X., Sun L., Li L., Deng Y., Wang J. (2021). Phloretin potentiates polymyxin E activity against gram-negative bacteria. Life Sci..

[141] Zhou H., Xu M., Guo W., Yao Z., Du X., Chen L., Sun Y., Shi S., Cao J., Zhou T. (2022). The Antibacterial Activity of Kaempferol Combined with Colistin against Colistin-Resistant Gram-Negative Bacteria. Microbiol. Spectr..

[142] Paprocka P., Mankowska A., Sklodowski K., Krol G., Wollny T., Lesiak A., Gluszek K., Savage P.B., Durnas B., Bucki R. (2022). Bactericidal Activity of Ceragenin in Combination with Ceftazidime, Levofloxacin, Co-Trimoxazole, and Colistin against the Opportunistic Pathogen *Stenotrophomonas maltophilia*. Pathogens.

[143] Yi K., Liu S., Liu P., Luo X., Zhao J., Yan F., Pan Y., Liu J., Zhai Y., Hu G. (2022). Synergistic antibacterial activity of tetrandrine combined with colistin against MCR-mediated colistin-resistant Salmonella. Biomed. Pharmacother..

[144] Shafiq M., Yao F., Bilal H., Rahman S.U., Zeng M., Ali I., Zeng Y., Li X., Yuan Y., Jiao X. (2022). Synergistic Activity of Tetrandrine and Colistin against mcr-1-Harboring *Escherichia coli*. Antibiotics.

[145] Dofini Magnini R., Pedinielli F., Vergalli J., Ouedraogo N., Remy S., Hilou A., Brunel J.M., Pages J.M., Davin-Regli A. (2023). *Acacia senegal* Budmunchiamines as a Potential Adjuvant for Rejuvenating Phenicol Activities towards *Escherichia coli*-Resistant Strains. Int. J. Mol. Sci..

[146] Li X., Song Y., Wang L., Kang G., Wang P., Yin H., Huang H. (2021). A Potential Combination Therapy of Berberine Hydrochloride With Antibiotics Against Multidrug-Resistant *Acinetobacter baumannii*. Front. Cell. Infect. Microbiol..

[147] Yap J.K.Y., Tan S.Y.Y., Tang S.Q., Thien V.K., Chan E.W.L. (2021). Synergistic Antibacterial Activity Between 1,4-Naphthoquinone and beta-Lactam Antibiotics Against Methicillin-Resistant *Staphylococcus aureus*. Microb. Drug Resist..

[148] Dwivedi G.R., Maurya A., Yadav D.K., Singh V., Khan F., Gupta M.K., Singh M., Darokar M.P., Srivastava S.K. (2019). Synergy of clavine alkaloid ‘chanoclavine’ with tetracycline against multi-drug-resistant *E. coli*. J. Biomol. Struct. Dyn..

[149] Wultanska D., Piotrowski M., Pituch H. (2020). The effect of berberine chloride and/or its combination with vancomycin on the growth, biofilm formation, and motility of Clostridioides difficile. Eur. J. Clin. Microbiol. Infect. Dis..

[150] Xie S., Li L., Zhan B., Shen X., Deng X., Tan W., Fang T. (2022). Pogostone Enhances the Antibacterial Activity of Colistin against MCR-1-Positive Bacteria by Inhibiting the Biological Function of MCR-1. Molecules.

[151] Dos Santos Barbosa C.R., Scherf J.R., de Freitas T.S., de Menezes I.R.A., Pereira R.L.S., Dos Santos J.F.S., de Jesus S.S.P., Lopes T.P., de Sousa Silveira Z., de Morais Oliveira-Tintino C.D. (2021). Effect of Carvacrol and Thymol on NorA efflux pump inhibition in multidrug-resistant (MDR) *Staphylococcus aureus* strains. J. Bioenerg. Biomembr..

[152] Lu X., Wang Y., Guo W., Zhang Z., Hu X., Nie T., Yang X., Li C., Wang X., Li X. (2023). Antibacterial Activity of an FtsZ Inhibitor Celastrol and Its Synergistic Effect with Vancomycin against Enterococci In Vitro and In Vivo. Microbiol. Spectr..

[153] Ruan Z., Cui J., He Z., Guo Y., Jia X., Huang X. (2020). Synergistic Effects from Combination of Cryptotanshinone and Fosfomycin Against Fosfomycin-Susceptible and Fosfomycin-Resistant *Staphylococcus aureus*. Infect. Drug Resist..

[154] Freitas P.R., de Araujo A.C.J., Barbosa C.R., Muniz D.F., Tintino S.R., Ribeiro-Filho J., Siqueira Junior J.P., Filho J.M.B., de Sousa G.R., Coutinho H.D.M. (2021). Inhibition of Efflux Pumps by Monoterpene (alpha-pinene) and Impact on *Staphylococcus aureus* Resistance to Tetracycline and Erythromycin. Curr. Drug Metab..

[155] Zhou Y., Liu B., Chu X., Su J., Xu L., Li L., Deng X., Li D., Lv Q., Wang J. (2022). Commercialized artemisinin derivatives combined with colistin protect against critical Gram-negative bacterial infection. Commun. Biol..

[156] Zhou Y., Lv X., Chen M., Guo Y., Ding R., Liu B., Deng X., Wang J. (2020). Characterization of Corosolic Acid as a KPC-2 Inhibitor That Increases the Susceptibility of KPC-2-Positive Bacteria to Carbapenems. Front. Pharmacol..

[157] Zhou Y., Guo Y., Wen Z., Ci X., Xia L., Wang Y., Deng X., Wang J. (2020). Isoalantolactone Enhances the Antimicrobial Activity of Penicillin G against *Staphylococcus aureus* by Inactivating beta-lactamase during Protein Translation. Pathogens.

[158] Hussein M., Allobawi R., Levou I., Blaskovich M.A.T., Rao G.G., Li J., Velkov T. (2022). Mechanisms Underlying Synergistic Killing of Polymyxin B in Combination with Cannabidiol against *Acinetobacter baumannii*: A Metabolomic Study. Pharmaceutics.

[159] Gallique M., Wei K., Maisuria V.B., Okshevsky M., McKay G., Nguyen D., Tufenkji N. (2021). Cranberry-Derived Proanthocyanidins Potentiate beta-Lactam Antibiotics against Resistant Bacteria. Appl. Environ. Microbiol..

[160] Sundaramoorthy N.S., Sivasubramanian A., Nagarajan S. (2020). Simultaneous inhibition of MarR by salicylate and efflux pumps by curcumin sensitizes colistin resistant clinical isolates of Enterobacteriaceae. Microb. Pathog..

[161] Guo Y., Lv X., Wang Y., Zhou Y., Lu N., Deng X., Wang J. (2020). Honokiol Restores Polymyxin Susceptibility to MCR-1-Positive Pathogens both In Vitro and In Vivo. Appl. Environ. Microbiol..

[162] Wang L., Zhang Y., Lin Y., Cao J., Xu C., Chen L., Wang Y., Sun Y., Zheng X., Liu Y. (2023). Resveratrol Increases Sensitivity of Clinical Colistin-Resistant *Pseudomonas aeruginosa* to Colistin In Vitro and In Vivo. Microbiol. Spectr..

[163] Yao Z., Feng L., Zhao Y., Zhang X., Chen L., Wang L., Zhang Y., Sun Y., Zhou T., Cao J. (2022). Thymol Increases Sensitivity of Clinical Col-R Gram-Negative Bacteria to Colistin. Microbiol. Spectr..

[164] Wang G., Li L., Wang X., Li X., Zhang Y., Yu J., Jiang J., You X., Xiong Y.Q. (2019). Hypericin enhances beta-lactam antibiotics activity by inhibiting sarA expression in methicillin-resistant *Staphylococcus aureus*. Acta Pharm. Sin. B.

[165] Shi C., Ma Y., Tian L., Li J., Qiao G., Liu C., Cao W., Liang C. (2022). Verbascoside: An Efficient and Safe Natural Antibacterial Adjuvant for Preventing Bacterial Contamination of Fresh Meat. Molecules.

[166] Ham S.Y., Kim H.S., Jo M.J., Lee J.H., Byun Y., Ko G.J., Park H.D. (2021). Combined Treatment of 6-Gingerol Analog and Tobramycin for Inhibiting *Pseudomonas aeruginosa* Infections. Microbiol. Spectr..

[167] Xu L., Zhou Y., Niu S., Liu Z., Zou Y., Yang Y., Feng H., Liu D., Niu X., Deng X. (2022). A novel inhibitor of monooxygenase reversed the activity of tetracyclines against tet(X3)/tet(X4)-positive bacteria. eBioMedicine.

[168] Song G., Zhou Y., Niu S., Deng X., Qiu J., Li L., Wang J. (2022). Nordihydroguaiaretic acid reverses the antibacterial activity of colistin against MCR-1-positive bacteria in vivo/in vitro by inhibiting MCR-1 activity and injuring the bacterial cell membrane. Phytomedicine.

[169] Liu Y., Yang K., Zhang H., Jia Y., Wang Z. (2020). Combating Antibiotic Tolerance Through Activating Bacterial Metabolism. Front. Microbiol..

[170] Meylan S., Porter C.B.M., Yang J.H., Belenky P., Gutierrez A., Lobritz M.A., Park J., Kim S.H., Moskowitz S.M., Collins J.J. (2017). Carbon Sources Tune Antibiotic Susceptibility in *Pseudomonas aeruginosa* via Tricarboxylic Acid Cycle Control. Cell Chem. Biol..

[171] Fan L., Pan Z., Liao X., Zhong Y., Guo J., Pang R., Chen X., Ye G., Su Y. (2023). Uracil restores susceptibility of methicillin-resistant *Staphylococcus aureus* to aminoglycosides through metabolic reprogramming. Front. Pharmacol..

[172] Kitzenberg D.A., Lee J.S., Mills K.B., Kim J.S., Liu L., Vazquez-Torres A., Colgan S.P., Kao D.J. (2022). Adenosine Awakens Metabolism to Enhance Growth-Independent Killing of Tolerant and Persister Bacteria across Multiple Classes of Antibiotics. mBio.

[173] Deng W., Fu T., Zhang Z., Jiang X., Xie J., Sun H., Hu P., Ren H., Zhou P., Liu Q. (2020). L-lysine potentiates aminoglycosides against *Acinetobacter baumannii* via regulation of proton motive force and antibiotics uptake. Emerg. Microbes Infect..

[174] Yang H., Zhou Y., Luo Q., Zhu C., Fang B. (2023). L-leucine increases the sensitivity of drug-resistant Salmonella to sarafloxacin by stimulating central carbon metabolism and increasing intracellular reactive oxygen species level. Front. Microbiol..

[175] Wang Z., Aweya J.J., Yao D., Zheng Z., Wang C., Zhao Y., Li S., Zhang Y. (2022). Taurine metabolism is modulated in Vibrio-infected Penaeus vannamei to shape shrimp antibacterial response and survival. Microbiome.

[176] Yang J., Yang X.L., Su Y.B., Peng X.X., Li H. (2021). Activation of the TCA Cycle to Provide Immune Protection in Zebrafish Immunized by High Magnesium-Prepared *Vibrio alginolyticus* Vaccine. Front. Immunol..

[177] Yang M.J., Xu D., Yang D.X., Li L., Peng X.X., Chen Z.G., Li H. (2020). Malate enhances survival of zebrafish against *Vibrio alginolyticus* infection in the same manner as taurine. Virulence.

[178] Crabbe A., Ostyn L., Staelens S., Rigauts C., Risseeuw M., Dhaenens M., Daled S., Van Acker H., Deforce D., Van Calenbergh S. (2019). Host metabolites stimulate the bacterial proton motive force to enhance the activity of aminoglycoside antibiotics. PLoS Pathog..

[179] Murima P., McKinney J.D., Pethe K. (2014). Targeting bacterial central metabolism for drug development. Chem. Biol..

[180] Gan B.H., Gaynord J., Rowe S.M., Deingruber T., Spring D.R. (2021). The multifaceted nature of antimicrobial peptides: Current synthetic chemistry approaches and future directions. Chem. Soc. Rev..

[181] Hancock R.E., Sahl H.G. (2006). Antimicrobial and host-defense peptides as new anti-infective therapeutic strategies. Nat. Biotechnol..

[182] Starr C.G., Ghimire J., Guha S., Hoffmann J.P., Wang Y., Sun L., Landreneau B.N., Kolansky Z.D., Kilanowski-Doroh I.M., Sammarco M.C. (2020). Synthetic molecular evolution of host cell-compatible, antimicrobial peptides effective against drug-resistant, biofilm-forming bacteria. Proc. Natl. Acad. Sci. USA.

[183] Chen C., Shi J., Wang D., Kong P., Wang Z., Liu Y. (2023). Antimicrobial peptides as promising antibiotic adjuvants to combat drug-resistant pathogens. Crit. Rev. Microbiol..

[184] Zhu Y., Hao W., Wang X., Ouyang J., Deng X., Yu H., Wang Y. (2022). Antimicrobial peptides, conventional antibiotics, and their synergistic utility for the treatment of drug-resistant infections. Med. Res. Rev..

[185] Mhlongo J.T., Waddad A.Y., Albericio F., de la Torre B.G. (2023). Antimicrobial Peptide Synergies for Fighting Infectious Diseases. Adv. Sci..

[186] Zharkova M.S., Orlov D.S., Golubeva O.Y., Chakchir O.B., Eliseev I.E., Grinchuk T.M., Shamova O.V. (2019). Application of Antimicrobial Peptides of the Innate Immune System in Combination With Conventional Antibiotics-A Novel Way to Combat Antibiotic Resistance?. Front. Cell. Infect. Microbiol..

[187] de Leeuw E., Li C., Zeng P., Li C., Diepeveen-de Buin M., Lu W.Y., Breukink E., Lu W. (2010). Functional interaction of human neutrophil peptide-1 with the cell wall precursor lipid II. FEBS Lett..

[188] Mathur H., Field D., Rea M.C., Cotter P.D., Hill C., Ross R.P. (2017). Bacteriocin-Antimicrobial Synergy: A Medical and Food Perspective. Front. Microbiol..

[189] Adeniji O.O., Nontongana N., Okoh J.C., Okoh A.I. (2022). The Potential of Antibiotics and Nanomaterial Combinations as Therapeutic Strategies in the Management of Multidrug-Resistant Infections: A Review. Int. J. Mol. Sci..

[190] Wang L., Hu C., Shao L. (2017). The antimicrobial activity of nanoparticles: Present situation and prospects for the future. Int. J. Nanomed..

[191] Raghunath A., Perumal E. (2017). Metal oxide nanoparticles as antimicrobial agents: A promise for the future. Int. J. Antimicrob. Agents.

[192] Kaiser K.G., Delattre V., Frost V.J., Buck G.W., Phu J.V., Fernandez T.G., Pavel I.E. (2023). Nanosilver: An Old Antibacterial Agent with Great Promise in the Fight against Antibiotic Resistance. Antibiotics.

[193] Chen C.W., Hsu C.Y., Lai S.M., Syu W.J., Wang T.Y., Lai P.S. (2014). Metal nanobullets for multidrug resistant bacteria and biofilms. Adv. Drug Deliv. Rev..

[194] Yin I.X., Zhang J., Zhao I.S., Mei M.L., Li Q., Chu C.H. (2020). The Antibacterial Mechanism of Silver Nanoparticles and Its Application in Dentistry. Int. J. Nanomed..

[195] Wahab M.A., Li L., Li H., Abdala A. (2021). Silver Nanoparticle-Based Nanocomposites for Combating Infectious Pathogens: Recent Advances and Future Prospects. Nanomaterials.

[196] Bruna T., Maldonado-Bravo F., Jara P., Caro N. (2021). Silver Nanoparticles and Their Antibacterial Applications. Int. J. Mol. Sci..

[197] Hutchings M.I., Truman A.W., Wilkinson B. (2019). Antibiotics: Past, present and future. Curr. Opin. Microbiol..

[198] Allahverdiyev A.M., Kon K.V., Abamor E.S., Bagirova M., Rafailovich M. (2011). Coping with antibiotic resistance: Combining nanoparticles with antibiotics and other antimicrobial agents. Expert Rev. Anti-Infect. Ther..

[199] Dove A.S., Dzurny D.I., Dees W.R., Qin N., Nunez Rodriguez C.C., Alt L.A., Ellward G.L., Best J.A., Rudawski N.G., Fujii K. (2022). Silver nanoparticles enhance the efficacy of aminoglycosides against antibiotic-resistant bacteria. Front. Microbiol..

[200] Adeniji O.O., Ojemaye M.O., Okoh A.I. (2022). Antibacterial Activity of Metallic Nanoparticles against Multidrug-Resistant Pathogens Isolated from Environmental Samples: Nanoparticles/Antibiotic Combination Therapy and Cytotoxicity Study. ACS Appl. Bio Mater..

[201] Gupta A., Mumtaz S., Li C.H., Hussain I., Rotello V.M. (2019). Combatting antibiotic-resistant bacteria using nanomaterials. Chem. Soc. Rev..

[202] Plank C. (2009). Nanomedicine: Silence the target. Nat. Nanotechnol..

[203] Allaker R.P., Ren G. (2008). Potential impact of nanotechnology on the control of infectious diseases. Trans. R. Soc. Trop. Med. Hyg..

[204] Wang Y., Ding X., Chen Y., Guo M., Zhang Y., Guo X., Gu H. (2016). Antibiotic-loaded, silver core-embedded mesoporous silica nanovehicles as a synergistic antibacterial agent for the treatment of drug-resistant infections. Biomaterials.

[205] Wang D., Jana D., Zhao Y. (2020). Metal-Organic Framework Derived Nanozymes in Biomedicine. Acc. Chem. Res..

[206] Wu W., Duan M., Shao S., Meng F., Qin Y., Zhang M. (2023). Recent advances in nanomaterial-mediated bacterial molecular action and their applications in wound therapy. Biomater. Sci..

[207] Ma T., Huang K., Cheng N. (2023). Recent Advances in Nanozyme-Mediated Strategies for Pathogen Detection and Control. Int. J. Mol. Sci..

[208] Ding X., Zhao Z., Zhang Y., Duan M., Liu C., Xu Y. (2023). Activity Regulating Strategies of Nanozymes for Biomedical Applications. Small.

[209] Liu Q., Zhang A., Wang R., Zhang Q., Cui D. (2021). A Review on Metal-and Metal Oxide-Based Nanozymes: Properties, Mechanisms, and Applications. Nano-Micro Lett..

[210] Yao H., Zhou R., Wang J., Wei Y., Li S., Zhang Z., Du X.D., Wu S., Shi J. (2023). Pathogen-Targeting Bimetallic Nanozymes as Ultrasonic-Augmented ROS Generator against Multidrug Resistant Bacterial Infection. Adv. Healthc. Mater..

[211] Baig M., Fatima A., Gao X., Farid A., Ajmal Khan M., Zia A.W., Wu H. (2022). Disrupting biofilm and eradicating bacteria by Ag-Fe_3_O_4_@MoS_2_ MNPs nanocomposite carrying enzyme and antibiotics. J. Control. Release.

[212] D’Accolti M., Soffritti I., Mazzacane S., Caselli E. (2021). Bacteriophages as a Potential 360-Degree Pathogen Control Strategy. Microorganisms.

[213] Abdelkader K., Gerstmans H., Saafan A., Dishisha T., Briers Y. (2019). The Preclinical and Clinical Progress of Bacteriophages and Their Lytic Enzymes: The Parts are Easier than the Whole. Viruses.

[214] Samoylova T.I., Braden T.D., Spencer J.A., Bartol F.F. (2017). Immunocontraception: Filamentous Bacteriophage as a Platform for Vaccine Development. Curr. Med. Chem..

[215] Samoylova T.I., Norris M.D., Samoylov A.M., Cochran A.M., Wolfe K.G., Petrenko V.A., Cox N.R. (2012). Infective and inactivated filamentous phage as carriers for immunogenic peptides. J. Virol. Methods.

[216] Shlezinger M., Friedman M., Houri-Haddad Y., Hazan R., Beyth N. (2019). Phages in a thermoreversible sustained-release formulation targeting E. faecalis in vitro and in vivo. PLoS ONE.

[217] Miedzybrodzki R., Borysowski J., Weber-Dabrowska B., Fortuna W., Letkiewicz S., Szufnarowski K., Pawelczyk Z., Rogoz P., Klak M., Wojtasik E. (2012). Clinical aspects of phage therapy. Adv. Virus Res..

[218] Sybesma W., Rohde C., Bardy P., Pirnay J.P., Cooper I., Caplin J., Chanishvili N., Coffey A., De Vos D., Expert Round Table on Acceptance and Re-Implementation of Bacteriophage Therapy (2018). Silk Route to the Acceptance and Re-Implementation of Bacteriophage Therapy-Part II. Antibiotics.

[219] Gordillo Altamirano F.L., Barr J.J. (2019). Phage Therapy in the Postantibiotic Era. Clin. Microbiol. Rev..

[220] Chaudhry W.N., Concepcion-Acevedo J., Park T., Andleeb S., Bull J.J., Levin B.R. (2017). Synergy and Order Effects of Antibiotics and Phages in Killing *Pseudomonas aeruginosa* Biofilms. PLoS ONE.

[221] Kamal F., Dennis J.J. (2015). Burkholderia cepacia complex Phage-Antibiotic Synergy (PAS): Antibiotics stimulate lytic phage activity. Appl. Environ. Microbiol..

[222] Oechslin F., Piccardi P., Mancini S., Gabard J., Moreillon P., Entenza J.M., Resch G., Que Y.A. (2017). Synergistic Interaction Between Phage Therapy and Antibiotics Clears *Pseudomonas aeruginosa* Infection in Endocarditis and Reduces Virulence. J. Infect. Dis..

[223] Shlezinger M., Coppenhagen-Glazer S., Gelman D., Beyth N., Hazan R. (2019). Eradication of Vancomycin-Resistant Enterococci by Combining Phage and Vancomycin. Viruses.

[224] Chan B.K., Sistrom M., Wertz J.E., Kortright K.E., Narayan D., Turner P.E. (2016). Phage selection restores antibiotic sensitivity in MDR *Pseudomonas aeruginosa*. Sci. Rep..

[225] Comeau A.M., Tetart F., Trojet S.N., Prere M.F., Krisch H.M. (2007). Phage-Antibiotic Synergy (PAS): Beta-lactam and quinolone antibiotics stimulate virulent phage growth. PLoS ONE.

[226] Morrisette T., Kebriaei R., Lev K.L., Morales S., Rybak M.J. (2020). Bacteriophage Therapeutics: A Primer for Clinicians on Phage-Antibiotic Combinations. Pharmacotherapy.

[227] Uchiyama J., Shigehisa R., Nasukawa T., Mizukami K., Takemura-Uchiyama I., Ujihara T., Murakami H., Imanishi I., Nishifuji K., Sakaguchi M. (2018). Piperacillin and ceftazidime produce the strongest synergistic phage-antibiotic effect in *Pseudomonas aeruginosa*. Arch. Virol..

[228] Ryan E.M., Alkawareek M.Y., Donnelly R.F., Gilmore B.F. (2012). Synergistic phage-antibiotic combinations for the control of *Escherichia coli* biofilms in vitro. FEMS Immunol. Med. Microbiol..

[229] Dickey J., Perrot V. (2019). Adjunct phage treatment enhances the effectiveness of low antibiotic concentration against *Staphylococcus aureus* biofilms in vitro. PLoS ONE.

[230] Segall A.M., Roach D.R., Strathdee S.A. (2019). Stronger together? Perspectives on phage-antibiotic synergy in clinical applications of phage therapy. Curr. Opin. Microbiol..

[231] Torres-Barcelo C., Arias-Sanchez F.I., Vasse M., Ramsayer J., Kaltz O., Hochberg M.E. (2014). A window of opportunity to control the bacterial pathogen *Pseudomonas aeruginosa* combining antibiotics and phages. PLoS ONE.

[232] Seed K.D., Yen M., Shapiro B.J., Hilaire I.J., Charles R.C., Teng J.E., Ivers L.C., Boncy J., Harris J.B., Camilli A. (2014). Evolutionary consequences of intra-patient phage predation on microbial populations. Elife.

[233] Ortega M.A., Guzman Merino A., Fraile-Martinez O., Recio-Ruiz J., Pekarek L., Guijarro L.G., Garcia-Honduvilla N., Alvarez-Mon M., Bujan J., Garcia-Gallego S. (2020). Dendrimers and Dendritic Materials: From Laboratory to Medical Practice in Infectious Diseases. Pharmaceutics.

[234] Stokes J.M., Yang K., Swanson K., Jin W., Cubillos-Ruiz A., Donghia N.M., MacNair C.R., French S., Carfrae L.A., Bloom-Ackermann Z. (2020). A Deep Learning Approach to Antibiotic Discovery. Cell.

[235] Liu Y., Yang K., Jia Y., Shi J., Tong Z., Fang D., Yang B., Su C., Li R., Xiao X. (2021). Gut microbiome alterations in high-fat-diet-fed mice are associated with antibiotic tolerance. Nat. Microbiol..

[236] Nolan A.C., Zeden M.S., Kviatkovski I., Campbell C., Urwin L., Corrigan R.M., Grundling A., O’Gara J.P. (2023). Purine Nucleosides Interfere with c-di-AMP Levels and Act as Adjuvants To Re-Sensitize MRSA To beta-Lactam Antibiotics. mBio.

[237] Zhou Y., Yong Y., Zhu C., Yang H., Fang B. (2022). Exogenous D-ribose promotes gentamicin treatment of several drug-resistant Salmonella. Front. Microbiol..

[238] Guo J., Pan Z., Fan L., Zhong Y., Pang R., Su Y. (2023). Effect of Three Different Amino Acids Plus Gentamicin Against Methicillin-Resistant Staphylococcus aureus. Infect. Drug Resist..

[239] Yong Y., Zhou Y., Liu K., Liu G., Wu L., Fang B. (2021). Exogenous Citrulline and Glutamine Contribute to Reverse the Resistance of Salmonella to Apramycin. Front. Microbiol..

[240] Guan Y., Lin M., Shen P., Zou Z. (2023). Alanine-mediated P cycle boosting enhances the killing efficiency of kasugamycin on antibiotic-resistant Xanthomonas oryzae. Front. Microbiol..

[241] Guan Y., Shen P., Lin M., Ye X. (2022). Exogenous Alanine Reverses the Bacterial Resistance to Zhongshengmycin with the Promotion of the P Cycle in Xanthomonas oryzae. Antibiotics.

[242] Zou Z., Lin M., Shen P., Guan Y. (2023). Alanine-Dependent TCA Cycle Promotion Restores the Zhongshengmycin-Susceptibility in Xanthomonas oryzae. Int. J. Mol. Sci..

[243] Tang X.K., Su Y.B., Ye H.Q., Dai Z.Y., Yi H., Yang K.X., Zhang T.T., Chen Z.G. (2021). Glucose-Potentiated Amikacin Killing of Cefoperazone/Sulbactam Resistant *Pseudomonas aeruginosa*. Front. Microbiol..

[244] Chen X.W., Wu J.H., Liu Y.L., Munang’andu H.M., Peng B. (2023). Fructose promotes ampicillin killing of antibiotic-resistant Streptococcus agalactiae. Virulence.

[245] Jiang M., Li X., Xie C.L., Chen P., Luo W., Lin C.X., Wang Q., Shu D.M., Luo C.L., Qu H. (2023). Fructose-enabled killing of antibiotic-resistant Salmonella enteritidis by gentamicin: Insight from reprogramming metabolomics. Int. J. Antimicrob. Agents.

[246] Song M., Liu Y., Huang X., Ding S., Wang Y., Shen J., Zhu K. (2020). A broad-spectrum antibiotic adjuvant reverses multidrug-resistant Gram-negative pathogens. Nat. Microbiol..

[247] Darwish R., Almaaytah A., Salama A. (2022). The design and evaluation of the antimicrobial activity of a novel conjugated penta-ultrashort antimicrobial peptide in combination with conventional antibiotics against sensitive and resistant strains of *S. aureus* and *E. coli.*. Res. Pharm. Sci..

[248] Chatupheeraphat C., Peamchai J., Luk-In S., Eiamphungporn W. (2023). Synergistic effect and antibiofilm activity of the antimicrobial peptide K11 with conventional antibiotics against multidrug-resistant and extensively drug-resistant *Klebsiella pneumoniae*. Front. Cell Infect. Microbiol..

[249] Ridyard K.E., Elsawy M., Mattrasingh D., Klein D., Strehmel J., Beaulieu C., Wong A., Overhage J. (2023). Synergy between Human Peptide LL-37 and Polymyxin B against Planktonic and Biofilm Cells of *Escherichia coli* and *Pseudomonas aeruginosa*. Antibiotics.

[250] Coya J.M., Fraile-Agreda V., de Tapia L., Garcia-Fojeda B., Saenz A., Bengoechea J.A., Kronqvist N., Johansson J., Casals C. (2022). Cooperative action of SP-A and its trimeric recombinant fragment with polymyxins against Gram-negative respiratory bacteria. Front. Immunol..

[251] Mumtaz S., Behera S., Joshi S., Mukhopadhyay K. (2022). Efficacy and Toxicity Studies of Novel alpha-MSH Analogues with Antibiofilm Action and beta-Lactam Resensitization Potential against MRSA. ACS Infect. Dis..

[252] Meng F., Nie T., Lyu Y., Lyu F., Bie X., Lu Y., Zhao M., Lu Z. (2022). Plantaricin A reverses resistance to ciprofloxacin of multidrug-resistant Staphylococcus aureus by inhibiting efflux pumps. Environ. Microbiol..

[253] Han W., Wei Z., Camesano T.A. (2022). New antimicrobial peptide-antibiotic combination strategy for *Pseudomonas aeruginosa* inactivation. Biointerphases.

[254] Sajid M.I., Lohan S., Kato S., Tiwari R.K. (2022). Combination of Amphiphilic Cyclic Peptide [R(4)W(4)] and Levofloxacin against Multidrug-Resistant Bacteria. Antibiotics.

[255] Mirzaei R., Alikhani M.Y., Arciola C.R., Sedighi I., Yousefimashouf R., Bagheri K.P. (2022). Prevention, inhibition, and degradation effects of melittin alone and in combination with vancomycin and rifampin against strong biofilm producer strains of methicillin-resistant *Staphylococcus epidermidis*. Biomed Pharmacother..

[256] Mirzaei R., Esmaeili Gouvarchin Ghaleh H., Ranjbar R. (2023). Antibiofilm effect of melittin alone and in combination with conventional antibiotics toward strong biofilm of MDR-MRSA and -*Pseudomonas aeruginosa*. Front. Microbiol..

[257] Mirzaei R., Alikhani M.Y., Arciola C.R., Sedighi I., Irajian G., Jamasbi E., Yousefimashouf R., Bagheri K.P. (2022). Highly Synergistic Effects of Melittin With Vancomycin and Rifampin Against Vancomycin and Rifampin Resistant Staphylococcus epidermidis. Front. Microbiol..

[258] Sacco F., Bitossi C., Casciaro B., Loffredo M.R., Fabiano G., Torrini L., Raponi F., Raponi G., Mangoni M.L. (2022). The Antimicrobial Peptide Esc(1-21) Synergizes with Colistin in Inhibiting the Growth and in Killing Multidrug Resistant Acinetobacter baumannii Strains. Antibiotics.

[259] Wesseling C.M.J., Wood T.M., Bertheussen K., Lok S., Martin N.I. (2021). Thrombin-Derived Peptides Potentiate the Activity of Gram-Positive-Specific Antibiotics against Gram-Negative Bacteria. Molecules.

[260] Zhu N., Zhong C., Liu T., Zhu Y., Gou S., Bao H., Yao J., Ni J. (2021). Newly designed antimicrobial peptides with potent bioactivity and enhanced cell selectivity prevent and reverse rifampin resistance in Gram-negative bacteria. Eur. J. Pharm. Sci..

[261] Peng J., Mishra B., Khader R., Felix L., Mylonakis E. (2021). Novel Cecropin-4 Derived Peptides against Methicillin-Resistant Staphylococcus aureus. Antibiotics.

[262] Morici P., Rizzato C., Ghelardi E., Rossolini G.M., Lupetti A. (2023). Sensitization of KPC and NDM *Klebsiella pneumoniae* To Rifampicin by the Human Lactoferrin-Derived Peptide hLF1-11. Microbiol. Spectr..

[263] Kebriaei R., Lev K.L., Shah R.M., Stamper K.C., Holger D.J., Morrisette T., Kunz Coyne A.J., Lehman S.M., Rybak M.J. (2022). Eradication of Biofilm-Mediated Methicillin-Resistant Staphylococcus aureus Infections In Vitro: Bacteriophage-Antibiotic Combination. Microbiol. Spectr..

[264] Luo J., Xie L., Yang M., Liu M., Li Q., Wang P., Fan J., Jin J., Luo C. (2023). Synergistic Antibacterial Effect of Phage pB3074 in Combination with Antibiotics Targeting Cell Wall against Multidrug-Resistant Acinetobacter baumannii In Vitro and Ex Vivo. Microbiol. Spectr..

[265] Luo J., Xie L., Liu M., Li Q., Wang P., Luo C. (2022). Bactericidal Synergism between Phage YC#06 and Antibiotics: A Combination Strategy to Target Multidrug-Resistant Acinetobacter baumannii In Vitro and In Vivo. Microbiol. Spectr..

[266] Vashisth M., Yashveer S., Jaglan A.B., Virmani N., Bera B.C., Vaid R.K., Anand T. (2022). Synergy of a virulent phage (phiAB182) with antibiotics leading to successful elimination of biofilms formed by MDR Acinetobacter baumannii. Can. J. Microbiol..

[267] Soontarach R., Nwabor O.F., Voravuthikunchai S.P. (2022). Interaction of lytic phage T1245 with antibiotics for enhancement of antibacterial and anti-biofilm efficacy against multidrug-resistant Acinetobacter baumannii. Biofouling.

[268] Lu H., Li Z., Elbaz A., Ni S.Q. (2023). Synergistic action of phages and lytic proteins with antibiotics: A combination strategy to target bacteria and biofilms. BMC Microbiol..

[269] Gorzynski M., De Ville K., Week T., Jaramillo T., Danelishvili L. (2023). Understanding the Phage-Host Interaction Mechanism toward Improving the Efficacy of Current Antibiotics in Mycobacterium abscessus. Biomedicines.

[270] Abdelsattar A.S., Eita M.A., Hammouda Z.K., Gouda S.M., Hakim T.A., Yakoup A.Y., Safwat A., El-Shibiny A. (2023). The Lytic Activity of Bacteriophage ZCSE9 against Salmonella enterica and Its Synergistic Effects with Kanamycin. Viruses.

[271] Zhao Y., Feng L., Zhou B., Zhang X., Yao Z., Wang L., Wang Z., Zhou T., Chen L. (2023). A newly isolated bacteriophage vB8388 and its synergistic effect with aminoglycosides against multi-drug resistant *Klebsiella oxytoca* strain FK-8388. Microb. Pathog..

[272] Van Nieuwenhuyse B., Van der Linden D., Chatzis O., Lood C., Wagemans J., Lavigne R., Schroven K., Paeshuyse J., de Magnee C., Sokal E. (2022). Bacteriophage-antibiotic combination therapy against extensively drug-resistant *Pseudomonas aeruginosa* infection to allow liver transplantation in a toddler. Nat. Commun..

[273] Gordillo Altamirano F.L., Kostoulias X., Subedi D., Korneev D., Peleg A.Y., Barr J.J. (2022). Phage-antibiotic combination is a superior treatment against Acinetobacter baumannii in a preclinical study. EBioMedicine.

[274] Simon K., Pier W., Kruttgen A., Horz H.P. (2021). Synergy between Phage Sb-1 and Oxacillin against Methicillin-Resistant Staphylococcus aureus. Antibiotics.

[275] Engeman E., Freyberger H.R., Corey B.W., Ward A.M., He Y., Nikolich M.P., Filippov A.A., Tyner S.D., Jacobs A.C. (2021). Synergistic Killing and Re-Sensitization of *Pseudomonas aeruginosa* to Antibiotics by Phage-Antibiotic Combination Treatment. Pharmaceuticals.

[276] Holger D.J., Lev K.L., Kebriaei R., Morrisette T., Shah R., Alexander J., Lehman S.M., Rybak M.J. (2022). Bacteriophage-antibiotic combination therapy for multidrug-resistant *Pseudomonas aeruginosa*: In vitro synergy testing. J. Appl. Microbiol..

[277] Wang Z., Cai R., Wang G., Guo Z., Liu X., Guan Y., Ji Y., Zhang H., Xi H., Zhao R. (2021). Combination Therapy of Phage vB_KpnM_P-KP2 and Gentamicin Combats Acute Pneumonia Caused by K47 Serotype *Klebsiella pneumoniae*. Front. Microbiol..

