# Supplementary material for: The Combination of Antibiotic and Non-Antibiotic Compounds Improves Antibiotic Efficacy against Multidrug-Resistant Bacteria
Source: Int J Mol Sci. 2023 Oct 23;24(20):15493. doi: 10.3390/ijms242015493 (PMC10607837; doi:10.3390/ijms242015493)
Supplement: Supplementary file 1 [file ijms-24-15493-s001.zip › ijms-2637033-supplementary.pdf]

Table S1. Representative synergistic combinations between metabolites with antibiotics against MDR bacteria

Metabolites	Combination with antibiotic	Antibiotic-resistance bacteria	Mechanism of action	Reference
Indole-3-acetic acid	Ciprofloxacin	MRSA、*E. coli*	Alter gut microbiota and promote energy metabolis	[235]
Glutamine	Ampicillin	Multidrug-resistant (MDR) bacteria	Stimulate influx of ampicillin, upregulate ompF, and inhibit biofilm	[115]
Thymine	Ciprofloxacin	Ciprofloxacin resistant *E. coli*	Upregulate bacterial metabolism	[113]
Adenosine	ampicillin, ceftriaxone, gentamicin	*S. aureus*	Inhibit guanosine tetraphosphate synthesis and the massive accumulation of ATP and GTP, generating proton motive force	[172]
Guanosine and xanthosine	Oxacillin and other β-lactam antibiotics	MRSA	Reduce the cyclic dinucleotide c-di-AMP	[236]
L-leucine	Sarafloxacin	Sarafloxacin-resistant *Salmonella*	Stimulate central carbon metabolism and increase intracellular ROS	[174]
D-ribose,	Gentamicin	Gentamicin-resistant *S. choleraesuis*	Activate central carbon metabolism	[237]
Threonine/glycine	Gentamicin	MRSA	Increase the activities of citrate synthase, isocitrate dehydrogenase and α-ketoglutarate dehydrogenase	[238]
Citrulline and Glutamine	Apramycin	Apramycin-resistant *Salmonella*	Promoted the TCA cycle, increase NADH production, increase PMF, increase apramycin accumulation	[239]
Alanine	Kasugamycin	Kasugamycin-resistant *Xanthomonas oryzae*	Promote the P cycle	[240]
Alanine	Zhongshengmycin	Zhongshengmycin-resistant *Xanthomonas oryzae*	Promote the P cycle and the TCA cycle	[241,242]

Glucose	Amikacin	Cefoperazone/Sulbactam-resistant *P. aeruginosa*	Promote PMF	[243]
Fructose	Ampicillin	Ampicillin-resistant *Streptococcus agalactiae*	Enhances ampicillin uptake and the expression of penicillin-binding proteins	[244]
Fructose	Gentamicin	Gentamicin-resistant *Salmonella*	Increase gentamicin intake	[245]
L-Serine	Macrolides	Macrolide-resistant *Streptococcus suis*	Inhibit intracellular H_2S production, reduce Fe-S cluster production, and restore the normal occurrence of the Fenton reaction in cells, increase ROS production	[110]

Table S2. Representative synergistic combinations between AMPs and antibiotics against MDR bacteria

**Amps**	**Combination with antibiotic**	**Antibiotic-resistance bacteria**	**Mechanism**	**Reference**
SLAP-S25	Colistin, tetracycline, vancomycin, rifampicin, ofloxacin, cefepime	MDR *E. coli* B2	Triggers membrane damage by binding to both lipopolysaccharide (LPS) and phosphatidylglycerol (PG)	[246]
Naprolyginine	Chloramphenicol	ESBL *E. coli* (BAA-3054)	---	[247]
K11	Chloramphenicol, meropenem, rifampicin, ceftazidime,	MDR and extensively drug-resistant *K. pneumoniae*	Inhibit biofilm	[248]
IL-37	Polymyxin B	*E. coli* MG1655 and *P. aeruginosa* PAO1	Inhibit biofilm	[249]
SP-A	Polymyxin B/colistin	*K. pneumoniae, Haemophilus influenzae, and P. aeruginosa*	Increase bacterial membrane permeabilization	[250]
Ana-10	β-Lactam antibiotics	MRSA	Membrane rupture	[251]
Plantaricin A	Ciprofloxacin	MDR *S. aureus*	Inhibited the function of the efflux pump	[252]

LL37	Vancomycin, azithromycin, polymyxin B, and colistin	*P. aeruginosa* strains PAO1 and PA103	Increase outer membrane permeabilization	[253]
Amphiphilic Cyclic Peptide [R4W4]	Levofloxacin	MRSA, carbapenem resistant *E. coli* and *P. aeruginosa*, imipenem resistant *K. pneumoniae*	Improve their potency to target drug-resistant bacteria	[254]
Melittin	Vancomycin and rifampin	MRSA	Downregulate the expression of biofilm-associated genes	[255]
Melittin	Gentamicin, ciprofloxacin, vancomycin, and rifampin	MDR-MRSA and MDR-*P. aeruginosa*	Inhibit biofilm	[256]
Melittin	Vancomycin and rifampin	Vancomycin and rifampin resistant *staphylococcus epidermidis*	Lysis of cell membranes	[257]
Frog-skin AMP Esc (1-21)	Colistin	MDR *A. baumannii*	Slow down growth and increase membrane-perturbing	[258]
C-terminal peptides	Erythromycin, rifampicin, novobiocin, vancomycin	MDR gram-negative strain	Increase outer membrane permeabilizing activity	[259]
L1GA5K	Rifampin	Rifampin-resistant *E. coli*	Increase outer membrane permeabilizing activity	[260]
Peptides C-18	Daptomycin	MRSA	Alter the membrane potential, increase membrane fluidity, and cause membrane breakage	[261]
Peptide hlf1-11	Rifampicin	MDR carbapenemase-producing *K. pneumoniae*	Transient loss of membrane potential and increase membrane permeability	[262]

Table S3. Representative synergistic combinations between phages and antibiotics against MDR bacteria

Phage	Combination with antibiotic	Antibiotic-resistance bacteria	Mechanism	Reference
Phage Sb-1	Daptomycin and ceftaroline	Vancomycin-intermediate, daptomycin-resistant *S. aureus*	Inhibit biofilm formation	[263]
Phage pb3074	Cefotaxime or meropenem	MDR *A. baumannii*	Target cell wall	[264]
Phage YC#06	Chloramphenicol, imipenem and cefotaxime	High-level MDR *A. baumannii*	Inhibit biofilm formation and remove mature biofilms	[265]
Phage φAB182	Colistin, polymyxin B, ceftazidime and cefotaxime	MDR *A. baumannii*	Eliminate of biofilm	[266]
Phage T1245	Ceftazidime, colistin, and meropenem	MDR *A. baumannii*	Disrupt of biofilm structure and cell morphology	[267
SSE1, SGF2 and SGF3	β-lactam antibiotics	*Shigella dysenteriae*	Target bacteria and biofilms	[268
Mycobacteriophage	Amikacin, cefoxitin, ciprofloxacin	*Mycobacterium abscessus*	Impair antibiotic efflux function	[269]
Bacteriophage ZCSE9	Kanamycin	*Salmonella enterica*	prevent bacterial growth	[270]
Bacteriophage vb8388	Aminoglycosides（gentamicin, amikacin, and tobramycin）	MDR *Klebsiella oxytoca* strain FK-8388	Enhance the anti-biofilm effect	[271]
Phage PNM	Colistin, aztreonam, and gentamycin	Drug-resistant *P. aeruginosa* sepsis	---	[272]
Phage øfg02	Ceftazidime	*A. baumannii* AB900	Disrupt cell morphology	[273]
Phage Sb-1	Oxacillin	MRSA	---	[274]
Phage cocktail PAM2H	Ceftazidime, ciprofloxacin, gentamicin, meropenem		Prevent mutations in genes encoding known phage receptors	[275]

Phage EM	Meropenem	MDR *Pseudomonas*	Decrease outer membrane vesicle production	[276]
Phage vb_kpnm_P-KP2	Gentamicin	K47 Serotype *K. pneumoniae*	---	[277]
